# Men's Representation in Behavioral Weight Loss Trials: A Systematic Review

**DOI:** 10.1111/obr.70170

**Published:** 2026-05-31

**Authors:** Casey P. Regan, Philip J. Morgan, Stephanie L. Hardacre, Levi Wade, Sherry L. Pagoto, Kate Hunt, James M. Knox, Ryan J. Drew, Myles D. Young

**Affiliations:** ^1^ School of Science, College of Engineering, Science, and Environment University of Newcastle Callaghan Australia; ^2^ Global Sport and Movement Collaborative The University of Newcastle Callaghan Australia; ^3^ School of Education, College of Human and Social Futures The University of Newcastle Callaghan Australia; ^4^ Centre of Excellence for Equity in Higher Education The University of Newcastle Callaghan Australia; ^5^ Department of Allied Health Sciences, College of Agriculture, Health, and Natural Resources University of Connecticut Storrs USA; ^6^ Institute for Social Marketing and Health, Faculty of Health Sciences and Sport University of Stirling Stirling UK; ^7^ School of Biomedical Sciences and Pharmacy, College of Health, Medicine and Wellbeing The University of Newcastle Callaghan Australia

**Keywords:** behavior change, diet, gender‐tailored, men, men's health, obesity, overweight, physical activity, randomized controlled trials

## Abstract

**Background:**

Historically, men have been underrepresented in behavioral weight loss intervention research, despite facing higher risks for obesity‐related diseases. Understanding this underrepresentation is critical for designing weight loss interventions that are both inclusive and effective.

**Objectives:**

The primary aim of this systematic review is to (1) examine the proportion of men participating in behavioral weight loss trials. Secondary research questions include (2) how often men are targeted as the primary population; (3) whether certain trial characteristics are associated with higher or lower male participation; and (4) in trials where men are underrepresented (< 40% of participants), how often researchers acknowledge this limitation or discuss implications for generalizability.

**Methods:**

A systematic search of SCOPUS, CENTRAL, and MEDLINE was conducted for randomized trials published in English between January 2010 and December 2022. Eligible studies recruited adults (≥ 18 years) into behavioral weight loss interventions using a randomized controlled design. Study characteristics associated with men's participation were examined using *t*‐tests and ANOVAs.

**Results:**

Of 57,497 participants across 257 trials, only 30% were men. Overall, 21% of studies recruited women only (*n* = 54) compared to 5% that recruited men only (*n* = 14). Men's participation was higher in trials targeting adults with a co‐occurring physical condition (36% vs. 28%). Of 189 trials recruiting both men and women, 76% recruited < 40% men, but only one‐third of these papers acknowledged this as a limitation.

**Conclusion:**

Men remain underrepresented in behavioral weight loss trials. To overcome this, strategies to improve men's uptake of existing interventions are needed, alongside the development of codesigned gender‐tailored approaches.

## 1 Introduction

Globally, the increasing rate of overweight and obesity in men is a major health concern. According to the Global Obesity Observatory, men with a body mass index (BMI) in the overweight or obesity range now outnumber those in the healthy weight range in 111 countries (60% of those with available data) [1]. While women are also impacted, men are less likely to attempt weight loss [2, 3, 4] and more likely to store fat abdominally [5], which increases their risk of chronic disease [6, 7, 8]. Improving these outcomes at scale will require political commitment to address obesogenic environments. However, engaging and effective weight loss interventions remain important for individuals seeking help.

Historically, men have been greatly underrepresented in behavioral weight loss research. In 2012, Pagoto and colleagues investigated the representation of men across 244 randomized controlled trials of lifestyle weight loss interventions conducted between 1999 and 2009 [9]. They found that men represented only 27% of participants. Of further concern, while 32% of studies recruited exclusively women, only 5% of studies focused exclusively on men. This imbalance is concerning because men are particularly susceptible to obesity‐related diseases such as type 2 diabetes [3] and cardiovascular disease [10]. As such, findings from interventions mostly tested with women may not generalize to men. Together, these factors highlight the need for gender‐specific approaches to behavioral weight loss interventions.

To increase the participation of men, Pagoto and colleagues called for researchers to increase men's engagement through an increased focus on gender‐tailored interventions designed specifically for men. In the time since this initial review was published, there has been significant growth in research related to obesity [11] and to behavioral weight loss interventions [12, 13, 14]. Importantly, several reviews have since evaluated the efficacy of gender‐tailored behavioral interventions across a range of outcomes, including physical activity [15], mental health [16], and weight loss [17, 18, 19]. There are also indications in various systematic reviews and meta‐analyses [15, 20, 21, 22] of an increase in the development and testing of gender‐tailored health promotion initiatives [23, 24, 25], which have demonstrated effectiveness at attracting participation and improving health outcomes among men. Considering these recent changes, we conducted this review to investigate the following research questions:

1. In behavioral weight loss trials, what proportion of participants are men?
2. How frequently do these trials target men as the primary population?
3. Are men more or less likely to participate in trials with certain characteristics?
4. In trials where men are underrepresented (i.e., < 40% of participants), how often do researchers identify this as a limitation or discuss the implications for study generalizability?

## 2 Methods

The conduct and reporting of this systematic review are in line with the Preferred Reporting for Systematic Reviews and Meta‐Analyses (PRISMA) [26]. A protocol was developed to guide the review process, but it was not preregistered prior to commencement.

### 2.1 Search Strategy

To expand on the previous review (which included studies up to 2009) [9], we searched SCOPUS, CENTRAL, and MEDLINE for eligible trials. Retrieved studies included the term “weight loss” plus the term “random*” and/or “RCT” in either the title, abstract, or keywords. Where possible, the following limits were applied: English language, journal article, randomized trial/randomized controlled trial, humans, and adults (see Supporting Information S1: File C for the complete search strategy used in each database).

### 2.2 Eligibility Screening and Data Extraction

Studies were included in the review if they met the following criteria:

1. *Participants*: The study population was adults aged 18 years and over;
2. *Interventions*: At least one study arm tested a behavioral intervention where participants received support to lose weight through changes in physical activity (including sedentary behavior) and/or dietary modification;
3. *Comparator*: The study used a randomized trial design comparing at least one intervention to a control/comparison group or another intervention;
4. *Outcome*: The primary aim of the study was weight loss, and the sample size was based on an explicit power calculation designed to detect changes in weight loss;
5. The study was published in a peer‐reviewed, English‐language journal between January 2010 and December 2022.

Reasons for exclusion were as follows:

1. The primary outcome related to weight gain prevention, weight loss maintenance, or reducing postpartum weight retention;
2. One or more study arms tested the effects of a pharmacological or surgical intervention on weight loss. However, studies testing the impact of a behavioral weight loss intervention prior to or postsurgery were not excluded if the surgery was conducted outside of the study timeframe.

### 2.3 Study Selection

We conducted the title and abstract screening plus the full‐text review through the online “Covidence” program. After demonstrating strong inter‐rater reliability in a random sample of 100 double‐coded papers (91% overall agreement, Cohen's kappa = 0.81), two reviewers (L.W. and S.H.) screened all retrieved articles for eligibility based on information contained in the title and abstract. Where the researchers could not confirm eligibility via the title and abstract, the full paper was retrieved. In the full‐text stage, articles were screened by three reviewers (M.D.Y., L.W., and S.H.) after demonstrating strong inter‐rater reliability in a random sample of 100 triple‐coded papers (93% overall agreement, Krippendorf's alpha = 0.82).

### 2.4 Development of Coding Criteria and Data Extraction

The study coding template was adapted from manuals used in two previous systematic reviews [9, 27]. After drafting the initial template, the research team pilot tested the data extraction process with a random selection of eligible articles and met to clarify ambiguous items. After repeating this process several times to improve the clarity of the coding criteria, a final version was confirmed for use in the review. For each study in the review, two authors (from a pool of 5) independently extracted the required data. All disagreements were resolved through discussion, with a third author acting as an independent tiebreaker where necessary (MDY). For an overview of the key data items extracted from each study, see Table 1.

**TABLE 1:** Study characteristic description and response options.

Characteristic	Description	Response options
Total participants	What was the total number of randomized participants?	☐ □ *N* =
Participating men	What was the total number of randomized men (or the proportion of all randomized participants if the number was not available)?	☐ □ *N* (preferred) = ☐ □ % (if *N* not reported) = ☐ □ Not reported
Participating women	What was the total number of randomized women (or the proportion of all randomized participants if the number was not available)?	☐ □ *N* (preferred) = ☐ □ % (if *N* not reported) = ☐ □ Not reported
Health condition	Did the study target people with a specific health condition other than overweight or obesity?	☐ □ Yes (please indicate) ☐ □ No
Eligibility status	Did the study explicitly state that eligibility was restricted to men or women?	☐ □ Yes (men only) ☐ □ Yes (women only) ☐ □ No
Comment on generalizability	Where < 40% of randomized participants were men, did the authors mention this as a potential limitation, or how it could impact on the generalizability of the findings?	☐ □ Yes (please describe) ☐ □ No
Targeted behaviors	Which behaviors did this intervention target?	☐ □ Physical activity only ☐ □ Diet only ☐ □ Both ☐ □ Other (please indicate)
Delivery mode	What was the primary intervention mode of delivery?	☐ □ Face‐to‐face (groups) ☐ □ Face‐to‐face (individual) ☐ □ Face‐to‐face (both) ☐ □ Distance (e.g., online) ☐ □ Other (please indicate)
Gender tailoring	Did the authors indicate that one or more of their interventions were gender‐tailored?	☐ □ Yes (tailored for women) ☐ □ Yes (tailored for men) ☐ □ No

### 2.5 Data Analysis

Analyses were conducted using SPSS version 27.0 (SPSS, Chicago, IL). Means for quantitative variables and percentages for categorical variables were calculated. Independent samples *t*‐tests and analyses of variance investigated whether any study factors were associated with men's involvement (e.g., examining whether the average proportion of men varied among studies testing one‐on‐one interventions, group interventions, or online/distance interventions). Because randomized trials prevent participants from self‐selecting into a preferred intervention arm, studies that compared multiple interventions that differed on a particular characteristic (e.g., group vs. online/distance) were removed from that analysis for that characteristic, as they could not be coded at the study level. Studies that explicitly excluded women or men from participation were also excluded from these analyses.

## 3 Results

### 3.1 Study Selection

As seen in Figure 1, the search strategy retrieved a total of 11,906 unique citations, which were imported into Covidence for screening. Most records were excluded during the title and abstract screening process (*n* = 10,791). Of the 1115 full‐text papers that were screened, 257 were deemed eligible for inclusion in the review (see Supporting Information S1: File A for a complete list of included studies).

**FIGURE 1:**
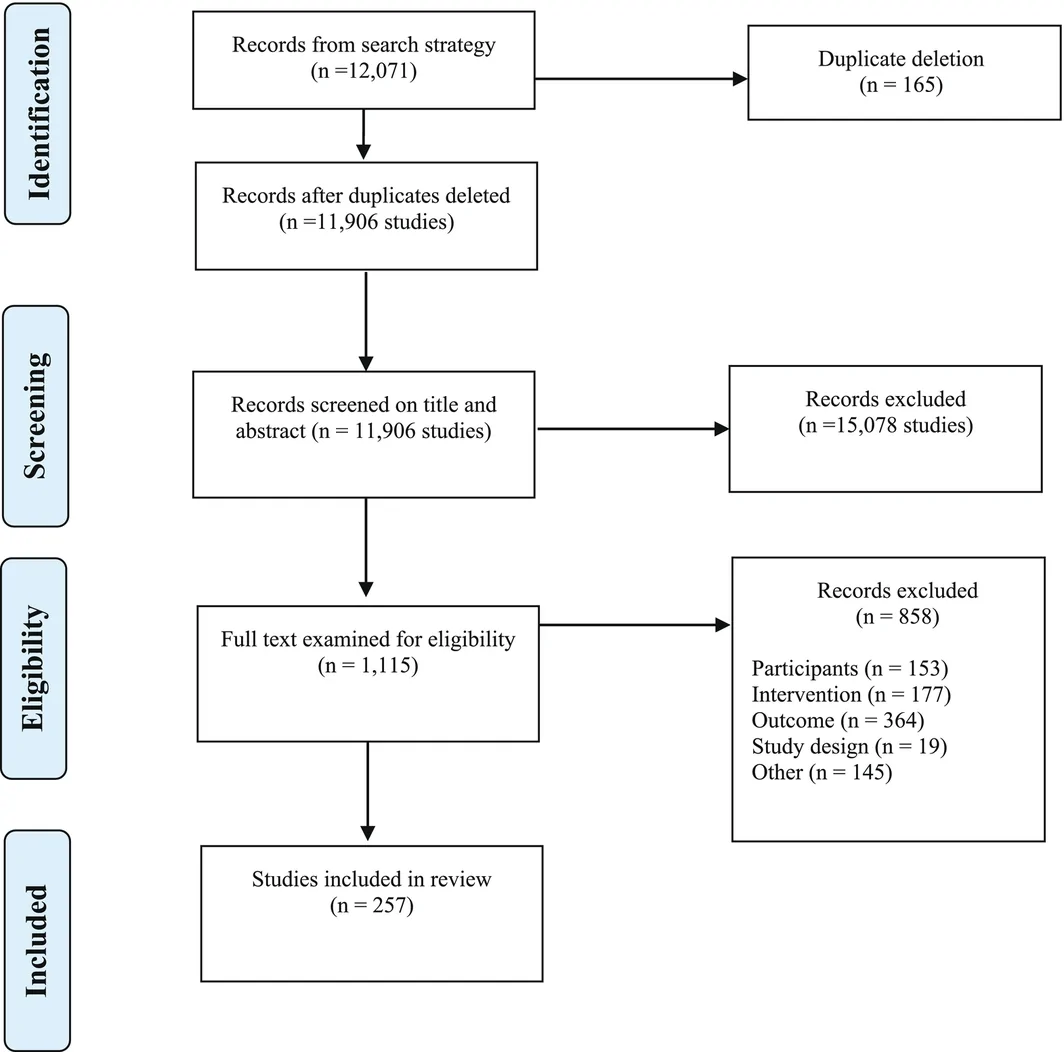
PRISMA flowchart of studies through the review process.

### 3.2 Study Characteristics

As seen in Table 2, most studies were conducted in the United States (58%), followed by the United Kingdom (8%) and Australia (5%). In 31% of studies, participants were required to have at least one co‐occurring physical health condition (e.g., type 2 diabetes and cancer), while 4% required a co‐occurring mental health condition (e.g., depression and binge eating disorder).

**TABLE 2:** Characteristics of included studies (*n* = 257).

Characteristic	*N* (%)	Characteristic	*N* (%)
Country		Co‐occurring condition (*N* = 87)	
United States	148 (58)	Mental health condition	10 (4)
United Kingdom	21 (8)	Physical health condition	79 (31)
Australia	13 (5)	Targeted behavior	
Japan	8 (3)	Physical activity and diet	218 (85)
Germany	9 (4)	Diet only	28 (11)
Iran	8 (3)	Physical activity only	1 (0)
Spain	7 (3)	Uncategorized^b^	10 (4)
New Zealand	6 (2)	Mode of delivery	
Brazil	6 (2)	Face‐to‐face (individual)	46 (18)
Other^a^	43 (17)	Face‐to‐face (group)	103 (40)
Year		Online/tech	40 (16)
2010–2013	60 (23)	Other distance	13 (5)
2014–2017	90 (35)	Uncategorized^b^	55 (21)
2018–2022	107 (42)	Gender‐tailored (*N* = 13)	
		For men	9 (4)
		For women	4 (2)

^a^ Netherlands (*n* = 4), Canada (*n* = 3), Sweden (*n* = 3), Finland (*n* = 2), Italy (*n* = 2), Norway (*n* = 2), South Korea (*n* = 2), Belgium (*n* = 1), China (*n* = 1), Czech Republic (*n* = 1), Denmark (*n* = 1), Latvia (*n* = 1), Malaysia (*n* = 1), Poland (*n* = 1), Saudi Arabia (*n* = 1), Singapore (*n* = 1), Taiwan (*n* = 1), Thailand (*n* = 1), Turkey (*n* = 1), Multinational (*n* = 1).

^b^ Study not categorized because characteristic differed between intervention arms (e.g., diet only vs. physical activity only).

Most trials tested weight loss interventions focused on both physical activity and dietary change (85%), followed by diet only (11%), and only one study focused on physical activity alone. Similarly, most studies tested interventions delivered face‐to‐face to groups (40%) or individuals (18%), with fewer testing programs delivered online (16%) or by other distance‐based means (e.g., phone calls, 5%). Thirteen studies (5%) tested interventions that were specifically “gender‐tailored” to appeal to either men (*n* = 9) or women (*n* = 4).

### 3.3 Research Question 1: In Behavioral Weight Loss Trials, What Proportion of Participants Are Men, and How Frequently Do These Trials Target Men as the Primary Population?

Overall, 57,497 participants were randomized into the 257 included trials. Across all studies, men represented 30% of participants (*n* = 17,222) and women represented 68.8% of participants (*n* = 39,553). Because some studies provided demographic data for completers only, the gender of 1.2% of randomized participants was unknown (*n* = 717). Only three studies reported genders outside the gender binary. In two of these studies, two participants in each self‐reported their gender as “other.” The remaining study included one gender variant/nonconforming participant. Overall, 21% of studies recruited women only (*n* = 54) compared to 5% that recruited men only (*n* = 14). Among the 189 studies that recruited both men and women (*n* = 47,884), 30.9% of participants were men (*n* = 14,814) and 67.7% were women (*n* = 32,409), with 1.4% of participants' gender not reported (*n* = 656).

### 3.4 Research Question 2: Were Men More or Less Likely to Participate in Trials With Certain Characteristics?

#### 3.4.1 Year of Publication

The involvement of men in weight loss RCTs has not changed significantly over time (*F*_2,186_ = 0.81, *p* = 0.446). Overall, men represented 33% of participants in studies published during 2010–2013, 32% in 2014–2017, and 29% in 2018–2022.

#### 3.4.2 United States Versus International

On average, men were significantly less likely to participate in weight loss studies conducted in the United States (26%) than in other countries (37%), *t*_187_ = −4.3, *p* < 0.001.

#### 3.4.3 Targeted Behavior(s)

Studies testing dietary weight loss interventions recruited a greater proportion of men on average (38%) than studies testing interventions targeting diet and physical activity together (30%), but the difference was not significant (*t*_182_ = 1.95, *p* = 0.053).

#### 3.4.4 Delivery Mode

On average, somewhat fewer men enrolled in studies testing interventions delivered face‐to‐face to groups (28%) or individuals (32%) compared to those delivered online (33%) or by other distance‐based modes (42%), but the difference was not significant (*F*_3,147_ = 1.740, *p* = 0.161).

#### 3.4.5 Co‐Occurring Physical Health Condition

Men were significantly more likely to participate in weight loss studies targeting adults with co‐occurring physical health conditions (36%) compared to those that did not (28%), *t*_187_ = 2.87, *p* = 0.005.

#### 3.4.6 Co‐Occurring Mental Health Condition

On average, weight loss studies for adults with co‐occurring mental health conditions recruited a smaller proportion of men (26%) compared to those that did not (31%), but the difference was not significant, *t*_187_ = −0.69, *p* = 0.49.

### 3.5 Research Question 3: Where Men Are Underrepresented, How Often Do Researchers Identify This as a Limitation or Discuss the Implications for Study Generalizability?

Overall, men represented < 40% of participants in 144 of the 189 studies (76%) where both men and women were eligible to participate. Of these, 34% of studies (*n* = 49) acknowledged this as a limitation, identified it as an area to improve in future research, or discussed the implications for the generalizability.

## 4 Discussion

This systematic review aimed to determine the current representation of men in randomized trials of behavioral weight loss interventions. Additionally, it aimed to investigate whether men were more or less likely to participate in trials with certain characteristics, and how often authors identified the underrepresentation of men as a limitation or discussed the implications for study generalizability. Findings revealed that across 189 studies, men represent only one‐third of participants in behavioral weight loss trials, and there is no trend towards this underrepresentation diminishing over time. Despite this underrepresentation, most studies did not acknowledge it as a limitation or describe its implications for generalizability. Men were more likely to participate in trials that were conducted outside the United States and in trials recruiting adults with a co‐occurring physical health condition other than obesity. Year of publication, mode of delivery, target behavior, and the presence of a mental health condition were not significantly associated with men's participation.

Across all studies, men represented just below one‐third of participants. These results are broadly consistent with Pagoto and colleagues' [9] previous review, which reported that men represented 27% of participants in behavioral weight loss trials conducted between 1999 and 2009. They're also generally aligned with findings from related reviews investigating men's participation in lifestyle weight loss interventions [22, 28, 29, 30]. For instance, Borek et al. [22] reported that men‐only group weight loss interventions were almost seven times less common than women‐only interventions, despite being twice as effective on average. This suggests that men‐only group weight loss interventions may improve both uptake and outcomes. Despite researchers calling for an increased availability of programs specifically for men, the current review found that women‐only studies were four times as frequent as studies that recruited men only (21% vs. 5%). This matches the proportion of men‐only studies reported by Pagoto and colleagues [9].

Both improving men's uptake of existing mixed‐gender behavioral weight loss programs as well as increasing the availability of men‐only and gender‐tailored interventions may be beneficial. Firstly, given that mixed‐gender interventions appear similarly effective for men and women when men do participate [31], enhancing men's uptake of these interventions through improved recruitment and messaging could be of benefit. For instance, recent research found no evidence of gender inequalities in trial invitation acceptance [30], suggesting that inviting more men to enroll could increase their participation [32]. Additionally, using gender‐sensitized messaging and language during recruitment may increase men's engagement and motivation to participate in weight loss interventions by better aligning with masculine identities and preferences [19, 20].

At the same time, the low availability of men‐only and gender‐tailored programs, despite evidence that such programs can enhance engagement and outcomes, indicates a need to expand these options. The perceived relevance or framing of the intervention may be inadequate or even off‐putting if not tailored to align with masculine identities [33, 34]. Interventions that reframe lifestyle behaviors to avoid conflict with traditional masculine ideals (e.g., limiting prescriptive, low‐intensity, or mixed‐gender formats) and, rather, emphasize masculine norms such as strength, autonomy, and competition may resonate more with men [35]. For example, gender‐tailored programs for men that are aligned with their preferences for flexibility [22, 36], simplicity, practicality, and the ability to be completed independently [29, 37], and for autonomy and control in health behaviors [20, 21, 33, 38], have been associated with higher engagement and improved physical health outcomes, including increased physical activity and weight loss [15, 21, 24, 39, 40, 41].

Taken together, this highlights that the underrepresentation of men in behavioral weight loss research has not changed over the last 25 years. The finding that trials from the United States had a significantly smaller mean proportion of men compared to international trials is particularly concerning given the increasing rates of obesity among men in the United States [42, 43, 44], and the nation's prominent role as a leader in weight loss research [11, 12, 45].

One possible explanation for men's underrepresentation in behavioral weight loss interventions is that these interventions focus heavily on teaching participants healthy habits relating to food shopping, meal planning, and meal preparation [46]. According to surveys performed by the Pew Research Center (2014–2016) [47] and National Health and Nutrition Examination Survey (NHANES; 2017–2020) [48], women are more likely to be the primary person in the household to do food shopping (80% vs. 20%) and meal planning and preparation (80% vs. 20%). To the extent that men are less likely to be involved in these tasks, intervention content that emphasizes such tasks may not be viewed as interesting or relevant to men. Just as men's participation in behavioral weight loss interventions has not changed over time, neither has men's participation in meal planning and preparation in the household. For example, the Pew Research Center and NHANES data are comparable to data from a US Department of Agriculture survey from 30 years ago that revealed only 23%–36% of men in households with a female counterpart reported to be involved in meal planning, shopping, and preparation [49]. This suggests that behavioral weight loss interventions may have become focused on women as a result of heteronormative gender roles that historically assigned women to take on grocery shopping and meal planning and preparation. Interestingly, research on traditional beliefs about gender roles finds that such beliefs among men are linked to less preventive care [50], worse self‐rated health, higher rates of chronic disease, and greater depressive symptomatology [51]. Further research is needed to understand how to design behavioral weight loss interventions that engage men and meet them where they are, alongside broader social efforts to redefine rigid gender norms of health‐related responsibilities within households.

Trials recruiting participants with a co‐occurring physical medical condition included a significantly larger proportion of men (36%) than those that did not (28%). One explanation for this is that these types of trials may be more prevalent in primary care settings, potentially increasing their appeal or accessibility to men. Men more often engage with healthcare services or initiate help‐seeking reactively, that is, in response to increasing severity of physical symptoms or illness, rather than intervening proactively for prevention [17, 33, 52, 53]. Men with existing physical health conditions may also be more likely to be referred to weight loss intervention trials through routine clinical care (compared to men who are not receiving such care), particularly in the context of managing cardiometabolic risk factors such as hypertension, type 2 diabetes, or heart disease. This referral trend may increase men's representation in such trials compared to those recruiting from community or general population samples. However, this also suggests that early prevention opportunities may be being missed for men, highlighting the need to develop strategies that engage men before disease onset. This finding may also reflect evidence that men are less likely to consider their weight a problem in isolation, with weight concerns often becoming more salient only when accompanied by physical comorbidities [54]. Compared to women, men are less likely to accurately perceive themselves as overweight, express dissatisfaction with their weight, be concerned about their weight, attempt weight loss, or feel motivated to lose weight [54]. The fact that men were not significantly underrepresented in trials also targeting mental health conditions is positive because it suggests that framing interventions around physical health and mental health does not reduce men's participation.

There were no significant differences in men's engagement by mode of intervention delivery (e.g., group‐based vs. individual). While nonsignificant, the patterns observed in this review, where the largest proportion of men were enrolled in programs without a face‐to‐face element and the lowest proportion were enrolled in group formats, were broadly consistent with those noted in Pagoto and colleagues' previous review [9]. Existing literature presents mixed findings regarding preferred modes of delivery of weight loss programs for men [55, 56]. Some studies suggest men prefer participating in programs in a group context among men with similar interests and/or demographics to themselves [40, 57, 58, 59, 60], a concept described as “contextual congruence,” while other studies indicate men prefer self‐directed individual programs, valuing autonomy and convenience, as opposed to a group context when attempting to lose weight [40, 61, 62, 63]. For instance, men may be more receptive to group programs that are exclusively for men, as indicated by Borek et al.'s [22] review findings. However, the current analysis was limited to those programs that recruited both men and women, where men were likely in the minority. This may have influenced their engagement, meaning group‐based formats may lose their appeal to men when delivered in mixed‐gender settings. The lack of a clear association between delivery mode and men's participation in the current review might therefore reveal their preferences for delivery mode may not be about format alone, but rather about how well the mode aligns with their identities and expectations.

Men were underrepresented (< 40% participation) in over three‐quarters (76%) of trials where both men and women could participate, yet only about one‐third (34%) of these studies acknowledged this as a limitation or discussed its implications for the generalizability of findings. Limited recognition of men's underrepresentation is consistent with previous reviews highlighting a clear gender‐blindness in weight loss and health behavior research [9, 27, 64]. As such, the underrepresentation of men in health promotion research will likely continue to be overlooked unless it is explicitly acknowledged as a research gap. Similarly, the suboptimal attention to men's participation across the included trials within the current review suggests that men's engagement in behavioral weight loss interventions is not yet seen as a priority issue within the field.

### 4.1 Strengths and Limitations

This systematic review has several strengths. We employed rigorous methods to comprehensively synthesize evidence from over 250 behavioral weight loss RCTs, providing an up‐to‐date overview of men's representation in such trials, and the factors associated with their participation. By examining whether authors acknowledged men's underrepresentation, this review offers insight into current research priorities in the field, showing that men's participation remains frequently overlooked. However, some limitations should be acknowledged. Firstly, the search was conducted in December 2022 and was not updated prior to submission due to resource constraints. However, given the large number of included trials (*n* = 257) and the stability of patterns in representation over time, we expect any omitted studies to have limited impact on the overall conclusions. Secondly, only studies published in English were included, which may limit generalizability across non‐English‐speaking contexts. Additionally, the reliance on published RCTs, while methodologically rigorous, excluded other relevant study designs, which may represent more emerging work in this area (e.g., quasi‐experimental studies, pilot interventions, or trials included in gray literature and non‐peer‐reviewed reports). Finally, the review did not include a formal risk of bias assessment, which is a common feature of systematic reviews. However, as our review focused on sample composition and factors related to men's participation rather than intervention efficacy, we omitted this for pragmatic reasons given it would not have meaningfully informed our findings.

### 4.2 Implications and Future Research Recommendations

Overall, the findings of this review have important implications for how future behavioral weight loss trials for men are designed and recruited for. Despite the concerning impact of overweight and obesity among men worldwide, the persistent underrepresentation of men demands urgent attention. Importantly, designing trials that are gender‐tailored to men's preferences and lived experiences (which evidence suggests can improve both engagement and health outcomes [4, 17, 29]) rather than solely relying on strategies to recruit more men into mixed‐gender programs will be key. This can be achieved through a codesign approach with men from diverse backgrounds (e.g., age, socioeconomic status, and cultural background) to ensure contextual and cultural relevance, particularly in relation to men's nuanced delivery preferences (e.g., format, setting, and messaging) and masculine ideals such as independence, autonomy, flexibility, and practicality. Using a codesign approach to tailor behavioral weight loss interventions for men may help reduce logistical barriers and avoid stereotyping. By doing so, such interventions could better integrate men's values and ultimately bridge the gap between evidence‐based practices and the types of weight loss trials men are willing to engage in [4, 56]. Additionally, using targeted gender‐sensitized themes, messaging, and language in recruitment to these trials may be an effective way to increase men's engagement and motivation to participate, by better aligning with masculine identities and preferences [19, 20]. Finally, embedding gender‐tailored weight loss programs within existing services used by men (e.g., sports clubs, occupational health, or routine GP visits) may offer increased opportunity for early intervention that appeals to men. These recommendations for future research are essential for achieving more equitable representation in behavioral weight loss research.

## 5 Conclusion

Men remain substantially underrepresented in behavioral weight loss trials, comprising only about one‐third of participants, with no clear improvement over the past 25 years. Our findings demonstrate that men are more likely to participate in trials recruiting participants with a co‐occurring physical medical condition (compared to those without), yet pertinent barriers to participation exist. The limited acknowledgment of men's underrepresentation in trial reporting risks perpetuating gender‐blind research practices. While increasing uptake of existing, nongender‐tailored behavioral weight loss interventions among men may offer some benefit in the short term, future research should also codesign gender‐tailored approaches that align with men's preferences and lived experiences to improve equitable representation. Addressing these issues is essential for developing behavioral weight loss interventions that meet the needs of all populations.

## Conflicts of Interest

The authors declare no conflicts of interest.

## Supporting information


**Data S1:** Supporting information.

## Data Availability

Data extracted from included studies are available from the authors upon reasonable request.
