# Supplementary material for: Men's Representation in Behavioral Weight Loss Trials: A Systematic Review
Source: Obes Rev. 2026 May 31;27(12):e70170. doi: 10.1111/obr.70170 (PMC13749309; doi:10.1111/obr.70170)
Supplement: Supplementary file 1 [file OBR-27-e70170-s001.pdf]

Obesity Reviews Journal

Men's Representation in Behavioural Weight Loss Trials: A Systematic Review

Casey P Regan [1,2], Philip J Morgan [2,3], Stephanie L Hardacre [4], Levi Wade [2,3], Sherry L Pagoto [5], Kate Hunt [6], James M Knox [1], Ryan J Drew [2,7], Myles D Young [1,2]

[1] School of Science, College of Engineering, Science, and Environment, University of Newcastle, Callaghan, Australia

[2] Global Sport and Movement Collaborative, The University of Newcastle, Callaghan, Australia

[3] School of Education, College of Human and Social Futures, The University of Newcastle, Callaghan, Australia

[4] Centre of Excellence for Equity in Higher Education, The University of Newcastle, Callaghan, Australia

[5] Department of Allied Health Sciences, College of Agriculture, Health, and Natural Resources, University of Connecticut, USA

[6] Institute for Social Marketing and Health, Faculty of Health Sciences and Sport, University of Stirling, Stirling, UK

[7] School of Biomedical Sciences and Pharmacy, College of Health, Medicine and Wellbeing, The University of Newcastle, Callaghan, Australia

Correspondence concerning this article should be addressed to Dr Casey Regan, Behavioural Sciences Building, 1 University Drive, Callaghan New South Wales 2308, Australia Email: Casey.Regan@newcastle.edu.au

Supplementary A: References of Included Studies

1. Abdi J, Eftekhar H, Mahmoodi M, Shojayzadeh D, Sadeghi R, Saber M. Effect of the Intervention Based on New Communication Technologies and the Social-Cognitive Theory on the Weight Control of the Employees with Overweight and Obesity. *Journal of research in health sciences*. 2015;15(4):256-261.
2. Abedishargh N, Farani AR, Gharraee B, Farahani H. Effectiveness of Internet-based Cognitive Behavioral Therapy in Weight Loss, Stress, Anxiety, and Depression via Virtual Group Therapy. *Iranian Journal of Psychiatry and Behavioral Sciences*. 2021;15(3)doi:10.5812/IJPBS.113096
3. Ackermann RT, Liss DT, Finch EA, et al. A Randomized Comparative Effectiveness Trial for Preventing Type 2 Diabetes. *American journal of public health*. 2015;105(11):2328-2334. doi:10.2105/AJPH.2015.302641
4. Ackermann RT, Sandy LG, Beauregard T, Coblitz M, Norton KL, Vojta D. A randomized comparative effectiveness trial of using cable television to deliver diabetes prevention programming. *Obesity (Silver Spring, Md)*. 2014;22(7):1601-1607. doi:10.1002/oby.20762
5. Aguiar EJ, Morgan PJ, Collins CE, Plotnikoff RC, Young MD, Callister R. Efficacy of the Type 2 Diabetes Prevention Using LifeStyle Education Program RCT. *American journal of preventive medicine*. 2016;50(3):353-364. doi:10.1016/j.amepre.2015.08.020
6. Ahern AL, Wheeler GM, Aveyard P, et al. Extended and standard duration weight-loss programme referrals for adults in primary care (WRAP): a randomised controlled trial. *Lancet (london, england)*. 2017;389(10085):2214-2225. doi:10.1016/S0140-6736(17)30647-5
7. Alamout MM, Rahmanian M, Aghamohammadi V, Mohammadi E, Nasiri K. Effectiveness of mindfulness based cognitive therapy on weight loss, improvement of hypertension and attentional bias to eating cues in overweight people. *International journal of nursing sciences*. 2020;doi:10.1016/j.ijnss.2019.12.010
8. Alghamdi RQ. A randomized controlled trial of a 12-week intensive lifestyle intervention program at a primary care obesity clinic for adults in western Saudi Arabia. *Saudi medical journal*. 2017;38(8):837-845. doi:10.15537/smj.2017.8.20553
9. Alick CL, Samuel-Hodge C, Ward D, Ammerman A, Rini C, Tate DF. Together Eating & Activity Matters (TEAM): results of a pilot randomized-clinical trial of a spousal support weight loss intervention for Black men. *Obesity science and practice*. 2018;4(1):62-75. doi:10.1002/osp4.142
10. Anderson AS, Chong HY, Craigie AM, et al. A novel approach to increasing community capacity for weight management a volunteer-delivered programme (ActWELL) initiated within breast screening clinics: a randomised controlled trial. *International Journal of Behavioral Nutrition & Physical Activity*. 2021;18(1):34. doi:https://dx.doi.org/10.1186/s12966-021-01099-7
11. Anderson AS, Craigie AM, Caswell S, et al. The impact of a bodyweight and physical activity intervention (BeWEL) initiated through a national colorectal cancer screening programme: randomised controlled trial. *BMJ (online)*. 2014;348doi:10.1136/bmj.g1823
12. Annesi JJ. Mediation of the relationship of behavioural treatment type and changes in psychological predictors of healthy eating by body satisfaction changes in women with obesity. *Obesity research & clinical practice*. 2017;11(1):97-107. doi:10.1016/j.orcp.2016.03.011
13. Annesi JJ. Relationship of Emotional Eating and Mood Changes Through Self-Regulation Within Three Behavioral Treatments for Obesity. *Psychological Reports*. 2019;122(5):1689-1706. doi:10.1177/0033294118795883
14. Annesi JJ, Johnson PH, Tennant GA, Porter KJ, McEwen KL. Weight Loss and the Prevention of Weight Regain: evaluation of a Treatment Model of Exercise Self-Regulation Generalizing to Controlled Eating. *Permanente journal*. 2016;20(3):15-146. doi:10.7812/TPP/15-146
15. Apiñaniz A, Cobos-Campos R, Sáez de Lafuente-Moríñigo A, et al. Effectiveness of randomized controlled trial of a mobile app to promote healthy lifestyle in obese and overweight patients. *Family practice*. 2019;36(6):699-705. doi:10.1093/fampra/cmz020
16. Appel LJ, Clark JM, Yeh HC, et al. Comparative effectiveness of weight-loss interventions in clinical practice. *New England journal of medicine*. 2011;365(21):1959-1968. doi:10.1056/NEJMoa1108660
17. Ard JD, Carson TL, Shikany JM, et al. Weight loss and improved metabolic outcomes amongst rural African American women in the Deep South: six-month outcomes from a community-based randomized trial. *Journal of internal medicine*. 2017;282(1):102-113. doi:10.1111/joim.12622
18. Assunção MCF, Gigante DP, Cardoso MA, Sartorelli DS, Santos IS. Randomized, controlled trial promotes physical activity and reduces consumption of sweets and sodium among overweight and obese adults. *Nutrition Research*. 2010;30(8):541-549. doi:10.1016/j.nutres.2010.07.006
19. Axley P, Kodali S, Kuo YF, et al. Text messaging approach improves weight loss in patients with nonalcoholic fatty liver disease: a randomized study. *Liver international*. 2018;38(5):924-931. doi:10.1111/liv.13622
20. Baer HJ, Rozenblum R, De La Cruz BA, et al. Effect of an Online Weight Management Program Integrated With Population Health Management on Weight Change: a Randomized Clinical Trial. *JAMA*.

2020;324(17):1737-1746. doi:10.1001/jama.2020.18977
21. Barnason S, Zimmerman L, Schulz P, Pullen C, Schuelke S. Weight management telehealth intervention for overweight and obese rural cardiac rehabilitation participants: a randomised trial. *Journal of clinical nursing*. 2019;28(9-10):1808-1818. doi:10.1111/jocn.14784
22. Beatty JA, Greene GW, Blissmer BJ, Delmonico MJ, Melanson KJ. Effects of a novel bites, steps and eating rate-focused weight loss randomised controlled trial intervention on body weight and eating behaviours. *Journal of human nutrition and dietetics*. 2020;33(3):330-341. doi:10.1111/jhn.12704
23. Becofsky K, Wing EJ, McCaffery J, Boudreau M, Wing RR. A Randomized Controlled Trial of a Behavioral Weight Loss Program for Human Immunodeficiency Virus-Infected Patients. *Clinical infectious diseases*. 2017;65(1):154-157. doi:10.1093/cid/cix238
24. Beeken RJ, Leurent B, Vickerstaff V, et al. A brief intervention for weight control based on habit-formation theory delivered through primary care: results from a randomised controlled trial. *International journal of obesity (2005)*. 2017;41(2):246-254. doi:10.1038/ijo.2016.206
25. Befort CA, VanWormer JJ, Desouza C, et al. Effect of Behavioral Therapy With In-Clinic or Telephone Group Visits vs In-Clinic Individual Visits on Weight Loss Among Patients With Obesity in Rural Clinical Practice: A Randomized Clinical Trial. *JAMA*. 2021;325(4):363-372. doi:https://dx.doi.org/10.1001/jama.2020.25855
26. Beleigoli A, Andrade AQ, Diniz MDF, Ribeiro AL. Personalized web-based weight loss behavior change program with and without dietitian online coaching for adults with overweight and obesity: randomized controlled trial. *Journal of medical Internet research*. 2020;22(11)doi:10.2196/17494
27. Bennett GG, Steinberg D, Askew S, et al. Effectiveness of an App and Provider Counseling for Obesity Treatment in Primary Care. *American journal of preventive medicine*. 2018;55(6):777-786. doi:10.1016/j.amepre.2018.07.005
28. Bennett GG, Warner ET, Glasgow RE, et al. Obesity treatment for socioeconomically disadvantaged patients in primary care practice. *Archives of internal medicine*. 2012;172(7):565-574. doi:10.1001/archinternmed.2012.1
29. Bertz F, Brekke HK, Ellegård L, Rasmussen KM, Wennergren M, Winkvist A. Diet and exercise weight-loss trial in lactating overweight and obese women. *American journal of clinical nutrition*. 2012;96(4):698-705. doi:10.3945/ajcn.112.040196
30. Blackman Carr LT, Samuel-Hodge CD, Ward DS, Evenson KR, Bangdiwala SI, Tate DF. Comparative effectiveness of a standard behavioral and physical activity enhanced behavioral weight loss intervention in Black women. *Women and Health*. 2020;60(6):676-691. doi:10.1080/03630242.2019.1700585
31. Bo S, Rahimi F, Goitre I, et al. Effects of Self-Conditioning Techniques (Self-Hypnosis) in Promoting Weight Loss in Patients with Severe Obesity: a Randomized Controlled Trial. *Obesity (Silver Spring, Md)*. 2018;26(9):1422-1429. doi:10.1002/oby.22262
32. Brame J, Centner C, Berg N, Bartlam M, Gollhofer A, Konig D. Effects of a 12-Week Web-Based Weight Loss Program for Adults With Overweight and Obesity on COVIDAge and Lifestyle-Related Cardiometabolic Risk Factors: A Randomized Controlled Trial. *Frontiers in Public Health*. 2022;10:868255. doi:https://dx.doi.org/10.3389/fpubh.2022.868255
33. Brown JC, Sarwer DB, Troxel AB, et al. A randomized trial of exercise and diet on body composition in survivors of breast cancer with overweight or obesity. *Breast Cancer Research and Treatment*. 2021;189(1):145-154. doi:10.1007/s10549-021-06284-7
34. Brown JC, Yung RL, Gobbie-Hurder A, et al. Randomized trial of a clinic-based weight loss intervention in cancer survivors. *Journal of cancer survivorship*. 2018;12(2):186-195. doi:10.1007/s11764-017-0657-5
35. Burke LE, Sereika SM, Parmanto B, et al. Effect of tailored, daily feedback with lifestyle self-monitoring on weight loss: The SMARTER randomized clinical trial. *Obesity*. 2022;30(1):75-84. doi:10.1002/oby.23321
36. Burke LE, Styn MA, Sereika SM, et al. Using mHealth technology to enhance self-monitoring for weight loss: a randomized trial. *American journal of preventive medicine*. 2012;43(1):20-26. doi:10.1016/j.amepre.2012.03.016
37. Buscemi J, Yurasek AM, Dennhardt AA, Martens MP, Murphy JG. A randomized trial of a brief intervention for obesity in college students. *Clinical obesity*. 2011;1(4-6):131-140. doi:10.1111/j.1758-8111.2011.00030.x
38. Butryn ML, Forman EM, Lowe MR, Gorin AA, Zhang F, Schaumberg K. Efficacy of environmental and acceptance-based enhancements to behavioral weight loss treatment: the ENACT trial. *Obesity (Silver Spring, Md)*. 2017;25(5):866-872. doi:10.1002/oby.21813
39. Cadmus-Bertram L, Nelson SH, Hartman S, Patterson RE, Parker BA, Pierce JP. Randomized trial of a phone- and web-based weight loss program for women at elevated breast cancer risk: the HELP study. *Journal of behavioral medicine*. 2016;39(4):551-559. doi:10.1007/s10865-016-9735-9
40. Cadmus-Bertram L, Wang JB, Patterson RE, Newman VA, Parker BA, Pierce JP. Web-based self-monitoring for weight loss among overweight/obese women at increased risk for breast cancer: the HELP pilot study. *Psycho-oncology*. 2013;22(8):1821-1828. doi:10.1002/pon.3219
41. Cai R, Chao J, Li D, Zhang M, Kong L, Wang Y. Effect of community-based lifestyle interventions on weight loss and cardiometabolic risk factors in obese elderly in China: a randomized controlled trial. *Experimental*

gerontology. 2019;128:110749. doi:10.1016/j.exger.2019.110749
42. Carnie A, Lin J, Aicher B, et al. Randomized trial of nutrition education added to internet-based information and exercise at the work place for weight loss in a racially diverse population of overweight women. *Nutrition and diabetes*. 2013;3(DECEMBER):1-7. doi:10.1038/nutd.2013.39
43. Catenacci VA, Ostendorf DM, Pan Z, et al. The Impact of Timing of Exercise Initiation on Weight Loss: an 18-Month Randomized Clinical Trial. *Obesity (Silver Spring, Md)*. 2019;27(11):1828-1838. doi:10.1002/oby.22624
44. Cayir Y, Akturk Z. Clinical effectiveness of group-based education with individual counseling in obese women. *Acta medica mediterranea*. 2014;30(4):903-906.
45. Chambliss HO, Huber RC, Finley CE, McDoniel SO, Kitzman-Ulrich H, Wilkinson WJ. Computerized self-monitoring and technology-assisted feedback for weight loss with and without an enhanced behavioral component. *Patient education and counseling*. 2011;85(3):375-382. doi:10.1016/j.pec.2010.12.024
46. Clark DO, Keith N, Weiner M, Xu H. Outcomes of an RCT of videoconference vs. in-person or in-clinic nutrition and exercise in midlife adults with obesity. *Obesity science and practice*. 2019;doi:10.1002/osp4.318
47. Coletta AM, Sanchez B, O'Connor A, et al. Alignment of diet prescription to genotype does not promote greater weight loss success in women with obesity participating in an exercise and weight loss program. *Obesity science and practice*. 2018;4(6):554-574. doi:10.1002/osp4.305
48. Collins CE, Morgan PJ, Jones P, et al. A 12-week commercial web-based weight-loss program for overweight and obese adults: randomized controlled trial comparing basic versus enhanced features. *Journal of medical Internet research*. 2012;14(2):e57. doi:10.2196/jmir.1980
49. Crane MM, Lutes LD, Ward DS, Bowling JM, Tate DF. A randomized trial testing the efficacy of a novel approach to weight loss among men with overweight and obesity. *Obesity (Silver Spring, Md)*. 2015;23(12):2398-2405. doi:10.1002/oby.21265
50. D'Amuri A, Sanz JM, Capatti E, et al. Effectiveness of high-intensity interval training for weight loss in adults with obesity: A randomised controlled non-inferiority trial. *BMJ Open Sport and Exercise Medicine*. 2021;7(3)doi:10.1136/bmjsem-2020-001021
51. Damschroder LJ, Lutes LD, Kirsh S, et al. Small-changes obesity treatment among veterans: 12-month outcomes. *American journal of preventive medicine*. 2014;47(5):541-553. doi:10.1016/j.amepre.2014.06.016
52. Das SK, Bukhari AS, Taetzsch AG, et al. Randomized trial of a novel lifestyle intervention compared with the Diabetes Prevention Program for weight loss in adult dependents of military service members. *American Journal of Clinical Nutrition*. 2021;114(4):1546-1559. doi:https://dx.doi.org/10.1093/ajcn/nqab259
53. Daubenmier J, Moran PJ, Kristeller J, et al. Effects of a mindfulness-based weight loss intervention in adults with obesity: a randomized clinical trial. *Obesity (Silver Spring, Md)*. 2016;24(4):794-804. doi:10.1002/oby.21396
54. de Freitas PP, de Menezes MC, Dos Santos LC, Pimenta AM, Ferreira AVM, Lopes ACS. The transtheoretical model is an effective weight management intervention: a randomized controlled trial. *BMC public health*. 2020;20(1):652. doi:10.1186/s12889-020-08796-1
55. de Menezes MC, Mingoti SA, Cardoso CS, Mendonça RD, Lopes ACS. Intervention based on Transtheoretical Model promotes anthropometric and nutritional improvements - A randomized controlled trial. *Eating behaviors*. 2015;17:37-44. doi:10.1016/j.eatbeh.2014.12.007
56. Demark-Wahnefried W, Rogers LQ, Gibson JT, et al. Randomized trial of weight loss in primary breast cancer: impact on body composition, circulating biomarkers and tumor characteristics. *International journal of cancer*. 2020;146(10):2784-2796. doi:10.1002/ijc.32637
57. Driver S, McShan E, Swank C, et al. Efficacy of the Diabetes Prevention Program Group Lifestyle Balance Program Modified for Individuals with TBI (GLB-TBI): results from a 12-month Randomized Controlled Trial. *Annals of Behavioral Medicine*. 2022;doi:10.1093/abm/kaac036
58. Duncan MJ, Fenton S, Brown WJ, et al. Efficacy of a Multi-component m-Health Weight-loss Intervention in Overweight and Obese Adults: a Randomised Controlled Trial. *International journal of environmental research and public health*. 2020;17(17)doi:10.3390/ijerph17176200
59. Dunn C, Olabode-Dada O, Whetstone L, et al. Using synchronous distance education to deliver a weight loss intervention: a randomized trial. *Obesity (Silver Spring, Md)*. 2016;24(1):44-50. doi:10.1002/oby.21315
60. Eakin EG, Reeves MM, Winkler E, et al. Six-month outcomes from living well with diabetes: a randomized trial of a telephone-delivered weight loss and physical activity intervention to improve glycemic control. *Annals of behavioral medicine*. 2013;46(2):193-203. doi:10.1007/s12160-013-9498-2
61. Eakin EG, Winkler EA, Dunstan DW, et al. Living well with diabetes: 24-month outcomes from a randomized trial of telephone-delivered weight loss and physical activity intervention to improve glycemic control. *Diabetes care*. 2014;37(8):2177-2185. doi:10.2337/dc13-2427
62. Eaton CB, Hartman SJ, Perzanowski E, et al. A Randomized Clinical Trial of a Tailored Lifestyle Intervention for Obese, Sedentary, Primary Care Patients. *Annals of family medicine*. 2016;14(4):311-319. doi:10.1370/afm.1952
63. Falkenhain K, Locke SR, Lowe DA, et al. Keyto app and device versus WW app on weight loss and

metabolic risk in adults with overweight or obesity: A randomized trial. *Obesity*. 2021;29(10):1606-1614. doi:https://dx.doi.org/10.1002/oby.23242
64. Faulconbridge LF, Driscoll CFB, Hopkins CM, et al. Combined Treatment for Obesity and Depression: A Pilot Study. *Obesity*. 2018;26(7):1144-1152. doi:10.1002/oby.22209
65. Fernández-Ruiz VE, Armero-Barranco D, Paniagua-Urbano JA, Sole-Agusti M, Ruiz-Sánchez A, Gómez-Marín J. Short-medium-long-term efficacy of interdisciplinary intervention against overweight and obesity: randomized controlled clinical trial. *International journal of nursing practice*. 2018;24(6):e12690. doi:10.1111/ijn.12690
66. Finkelstein EA, Tham KW, Haaland BA, Sahasranaman A. Applying economic incentives to increase effectiveness of an outpatient weight loss program (TRIO) - A randomized controlled trial. *Social science & medicine (1982)*. 2017;185:63-70. doi:10.1016/j.socscimed.2017.05.030
67. Fischer HH, Fischer IP, Pereira RI, Furniss AL, Rozwadowski JM, Moore SL. Text Message Support for Weight Loss in Patients With Prediabetes: a Randomized Clinical Trial. *Diabetes care*. 2016;
68. Fitzgibbon ML, Stolley MR, Schiffer L, Sharp LK, Singh V, Dyer A. Obesity reduction black intervention trial (ORBIT): 18-month results. *Obesity (Silver Spring, Md)*. 2010;18(12):2317-2325. doi:10.1038/oby.2010.47
69. Fitzgibbon ML, Tussing-Humphreys L, Schiffer L, et al. Fit and Strong! Plus: twelve and eighteen month follow-up results for a comparative effectiveness trial among overweight/obese older adults with osteoarthritis. *Preventive medicine*. 2020;141doi:10.1016/j.ypmed.2020.106267
70. Forman EM, Goldstein SP, Crochiere RJ, et al. Randomized controlled trial of OnTrack, a just-in-time adaptive intervention designed to enhance weight loss. *Translational behavioral medicine*. 2019;9(6):989-1001. doi:10.1093/tbm/ibz137
71. Foster GD, Wyatt HR, Hill JO, et al. Weight and metabolic outcomes after 2 years on a low-carbohydrate versus low-fat diet: a randomized trial. *Annals of internal medicine*. 2010;153(3):147-157. doi:10.7326/0003-4819-153-3-201008030-00005
72. Foster-Schubert KE, Alfano CM, Duggan CR, et al. Effect of diet and exercise, alone or combined, on weight and body composition in overweight-to-obese postmenopausal women. *Obesity (Silver Spring, Md)*. 2012;20(8):1628-1638. doi:10.1038/oby.2011.76
73. Froehlich-Grobe K, Betts AC, Driver SJ, et al. Group Lifestyle Balance Adapted for Individuals With Impaired Mobility: outcomes for 6-Month RCT and Combined Groups at 12 Months. *American journal of preventive medicine*. 2020;59(6):805-817. doi:10.1016/j.amepre.2020.06.023
74. Fukuoka Y, Gay CL, Joiner KL, Vittinghoff E. A Novel Diabetes Prevention Intervention Using a Mobile App: a Randomized Controlled Trial With Overweight Adults at Risk. *American journal of preventive medicine*. 2015;49(2):223-237. doi:10.1016/j.amepre.2015.01.003
75. Gallagher R, Kirkness A, Zelestis E, et al. A randomised trial of a weight loss intervention for overweight and obese people diagnosed with coronary heart disease and/or type 2 diabetes. *Annals of behavioral medicine*. 2012;44(1):119-128. doi:10.1007/s12160-012-9369-2
76. Gardner CD, Trepanowski JF, Del Gobbo LC, et al. Effect of Low-Fat vs Low-Carbohydrate Diet on 12-Month Weight Loss in Overweight Adults and the Association With Genotype Pattern or Insulin Secretion: the DIETFITS Randomized Clinical Trial. *JAMA*. 2018;319(7):667-679. doi:10.1001/jama.2018.0245
77. Geiker NRW, Ritz C, Pedersen SD, Larsen TM, Hill JO, Astrup A. A weight-loss program adapted to the menstrual cycle increases weight loss in healthy, overweight, premenopausal women: a 6-mo randomized controlled trial. *American journal of clinical nutrition*. 2016;104(1):15-20. doi:10.3945/ajcn.115.126565
78. Glanz K, Shaw PA, Kwong PL, et al. Effect of Financial Incentives and Environmental Strategies on Weight Loss in the Healthy Weigh Study: A Randomized Clinical Trial. *JAMA Network Open*. 2021;4(9):e2124132. doi:https://dx.doi.org/10.1001/jamanetworkopen.2021.24132
79. Godino JG, Golaszewski NM, Norman GJ, et al. Text messaging and brief phone calls for weight loss in overweight and obese English- and Spanish-speaking adults: a 1-year, parallel-group, randomized controlled trial. *PLoS medicine*. 2019;16(9):e1002917. doi:10.1371/journal.pmed.1002917
80. Godino JG, Merchant G, Norman GJ, et al. Using social and mobile tools for weight loss in overweight and obese young adults (Project SMART): a 2 year, parallel-group, randomised, controlled trial. *The lancet Diabetes & endocrinology*. 2016;4(9):747-755. doi:10.1016/S2213-8587(16)30105-X
81. Goodpaster BH, Delany JP, Otto AD, et al. Effects of diet and physical activity interventions on weight loss and cardiometabolic risk factors in severely obese adults: a randomized trial. *JAMA*. 2010;304(16):1795-1802. doi:10.1001/jama.2010.1505
82. Gorin AA, Powers TA, Gettens K, et al. A randomized controlled trial of a theory-based weight-loss program for couples. *Health psychology*. 2020;39(2):137-146. doi:10.1037/hea0000808
83. Green BB, Anderson ML, Cook AJ, et al. e-Care for heart wellness: a feasibility trial to decrease blood pressure and cardiovascular risk. *American journal of preventive medicine*. 2014;46(4):368-377. doi:10.1016/j.amepre.2013.11.009
84. Green CA, Yarborough BJ, Leo MC, et al. The STRIDE weight loss and lifestyle intervention for individuals taking antipsychotic medications: a randomized trial. *American journal of psychiatry*. 2015;172(1):71-81. doi:10.1176/appi.ajp.2014.14020173

85. Greenlee HA, Crew KD, Mata JM, et al. A pilot randomized controlled trial of a commercial diet and exercise weight loss program in minority breast cancer survivors. *Obesity (Silver Spring, Md)*. 2013;21(1):65-76. doi:10.1002/oby.20245
86. Haggerty AF, Huepenbecker S, Sarwer DB, et al. The use of novel technology-based weight loss interventions for obese women with endometrial hyperplasia and cancer. *Gynecologic oncology*. 2016;140(2):239-244. doi:10.1016/j.ygyno.2015.11.033
87. Haire-Joshu D, Schwarz CD, Steger-May K, et al. A Randomized Trial of Weight Change in a National Home Visiting Program. *American journal of preventive medicine*. 2018;54(3):341-351. doi:10.1016/j.amepre.2017.12.012
88. Hajek P, Przulj D, Pesola F, et al. A randomised controlled trial of the 5:2 diet. *PLoS ONE [Electronic Resource]*. 2021;16(11):e0258853. doi:https://dx.doi.org/10.1371/journal.pone.0258853
89. Hales S, Turner-McGrievy GM, Wilcox S, et al. Social networks for improving healthy weight loss behaviors for overweight and obese adults: a randomized clinical trial of the social pounds off digitally (Social POD) mobile app. *International journal of medical informatics*. 2016;94:81-90. doi:10.1016/j.ijmedinf.2016.07.003
90. Hardcastle SJ, Taylor AH, Bailey MP, Harley RA, Hagger MS. Effectiveness of a motivational interviewing intervention on weight loss, physical activity and cardiovascular disease risk factors: a randomised controlled trial with a 12-month post-intervention follow-up. *International journal of behavioral nutrition and physical activity*. 2013;10:40-55. doi:10.1186/1479-5868-10-40
91. Harrigan M, Cartmel B, Loftfield E, et al. Randomized Trial Comparing Telephone Versus In-Person Weight Loss Counseling on Body Composition and Circulating Biomarkers in Women Treated for Breast Cancer: the Lifestyle, Exercise, and Nutrition (LEAN) Study. *Journal of clinical oncology*. 2016;34(7):669-676. doi:10.1200/JCO.2015.61.6375
92. Harvey-Berino J, West D, Krukowski R, et al. Internet delivered behavioral obesity treatment. *Preventive medicine*. 2010;51(2):123-128. doi:10.1016/j.ypmed.2010.04.018
93. Hayes JF, Balantekin KN, Graham AK, Strube MJ, Bickel WK, Wilfley DE. Implementation intentions for weight loss in college students with overweight and obesity: A proof-of-concept randomized controlled trial. *Translational Behavioral Medicine*. 2021;11(2):359-368. doi:10.1093/tbm/ibaa038
94. Heideman WH, de Wit M, Middelkoop BJ, et al. Diabetes risk reduction in overweight first degree relatives of type 2 diabetes patients: effects of a low-intensive lifestyle education program (DiAlert) A randomized controlled trial. *Patient education and counseling*. 2015;98(4):476-483. doi:10.1016/j.pec.2014.12.008
95. Hernández-Reyes A, Cámara-Martos F, Molina-Luque R, Romero-Saldaña M, Molina-Recio G, Moreno-Rojas R. Changes in body composition with a hypocaloric diet combined with sedentary, moderate and high-intense physical activity: a randomized controlled trial. *BMC women's health*. 2019;19(1):167. doi:10.1186/s12905-019-0864-5
96. Hernández-Reyes A, Cámara-Martos F, Vidal Á, Molina-Luque R, Moreno-Rojas R. Effects of Self-Weighing During Weight Loss Treatment: A 6-Month Randomized Controlled Trial. *Frontiers in Psychology*. 2020;11doi:10.3389/fpsyg.2020.00397
97. Hersey JC, Khavjou O, Strange LB, et al. The efficacy and cost-effectiveness of a community weight management intervention: a randomized controlled trial of the health weight management demonstration. *Preventive medicine*. 2012;54(1):42-49. doi:10.1016/j.ypmed.2011.09.018
98. Hilbert A, Neuhaus P, Kohler N, Petroff D, Hay P, Hubner C. Group cognitive remediation therapy prior to behavioral weight loss treatment for adults with severe obesity: A randomized clinical trial (CRT study). *Journal of Consulting & Clinical Psychology*. 2021;89(8):695-706. doi:https://dx.doi.org/10.1037/ccp0000668
99. Hitoshi F, Tetsuji Y, Itsuro Y, Shunsaku M. A Randomized Controlled Trial to Evaluate the Effects of Health Guidance with Video Call as Compared to Face-to-Face Health Guidance. *International medical journal*. 2017;24(2):186-191.
100. Holliday A, Burgin A, Fernandez EV, Fenton SAM, Thielecke F, Blannin AK. Points-based physical activity: a novel approach to facilitate changes in body composition in inactive women with overweight and obesity. *BMC public health*. 2018;18(1):261. doi:10.1186/s12889-018-5125-2
101. Holmes VA, Draffin CR, Patterson CC, et al. Postnatal Lifestyle Intervention for Overweight Women With Previous Gestational Diabetes: a Randomized Controlled Trial. *Journal of clinical endocrinology and metabolism*. 2018;103(7):2478-2487. doi:10.1210/jc.2017-02654
102. Hsu YI, Chen YC, Lee CL, Chang NJ. Effects of diet control and telemedicine-based resistance exercise intervention on patients with obesity and knee osteoarthritis: A randomized control trial. *International Journal of Environmental Research and Public Health*. 2021;18(15)doi:10.3390/ijerph18157744
103. Hunt K, Wyke S, Gray CM, et al. A gender-sensitised weight loss and healthy living programme for overweight and obese men delivered by Scottish Premier League football clubs (FFIT): a pragmatic randomised controlled trial. *Lancet*. 2014;doi:10.1016/S0140-6736(13)62420-4
104. Hurkmans E, Matthys C, Bogaerts A, Scheys L, Devloo K, Seghers J. Face-to-face versus mobile versus blended weight loss program: Randomized clinical trial. *JMIR mhealth and uhealth*. 2018;6(1)doi:10.2196/mhealth.7713
105. Huseinovic E, Bertz F, Leu Agelii M, Johansson EH, Winkvist A, Brekke HK. Effectiveness of a weight loss

intervention in postpartum women: results from a randomized controlled trial in primary health care. *American journal of clinical nutrition*. 2016;104(2):362-370. doi:10.3945/ajcn.116.135673

106. Imanaka M, Ando M, Kitamura T, Kawamura T. Effectiveness of web-based self-disclosure peer-to-peer support for weight loss: randomized controlled trial. *Journal of medical Internet research*. 2013;15(7):e136. doi:10.2196/jmir.2405

107. Innes AQ, Thomson G, Cotter M, King JA, Vollaard NBJ, Kelly BM. Evaluating differences in the clinical impact of a free online weight loss programme, a resource-intensive commercial weight loss programme and an active control condition: a parallel randomised controlled trial. *BMC public health*. 2019;19(1):1732. doi:10.1186/s12889-019-8061-x

108. Iqbal N, Vetter ML, Moore RH, et al. Effects of a low-intensity intervention that prescribed a low-carbohydrate vs. a low-fat diet in obese, diabetic participants. *Obesity (Silver Spring, Md)*. 2010;18(9):1733-1738. doi:10.1038/oby.2009.460

109. Jakicic JM, Davis KK, Rogers RJ, et al. Effect of Wearable Technology Combined with a Lifestyle Intervention on Long-term Weight Loss: the IDEA Randomized Clinical Trial. *Obstetrical & gynecological survey*. 2017;72(1):27-28. doi:10.1097/01.ogx.0000511086.69375.3b

110. Jakicic JM, Otto AD, Lang W, et al. The effect of physical activity on 18-month weight change in overweight adults. *Obesity (Silver Spring, Md)*. 2011;19(1):100-109. doi:10.1038/oby.2010.122

111. Jakicic JM, Rickman AD, Lang W, et al. Time-based physical activity interventions for weight loss: a randomized trial. *Medicine and science in sports and exercise*. 2015;47(5):1061-1069. doi:10.1249/MSS.0000000000000482

112. Jakicic JM, Tate DF, Lang W, et al. Effect of a stepped-care intervention approach on weight loss in adults: a randomized clinical trial. *JAMA*. 2012;307(24):2617-2626. doi:10.1001/jama.2012.6866

113. Jamal SN, Moy FM, Mohamed MNA, Mukhtar F. Effectiveness of a group support lifestyle modification (GSLiM) programme among obese adults in workplace: a randomised controlled trial. *PloS one*. 2016;11(8)(no pagination)doi:10.1371/journal.pone.0160343

114. Jane M, Hagger M, Foster J, Ho S, Kane R, Pal S. Effects of a weight management program delivered by social media on weight and metabolic syndrome risk factors in overweight and obese adults: A randomised controlled trial. *PloS one*. 2017;12(6)doi:10.1371/journal.pone.0178326

115. Jebb SA, Ahern AL, Olson AD, et al. Primary care referral to a commercial provider for weight loss treatment versus standard care: a randomised controlled trial. *Lancet (london, england)*. 2011;378(9801):1485-1492. doi:10.1016/S0140-6736(11)61344-5

116. Jiskoot G, Timman R, Beerthuizen A, Dietz de Loos A, Busschbach J, Laven J. Weight Reduction Through a Cognitive Behavioral Therapy Lifestyle Intervention in PCOS: the Primary Outcome of a Randomized Controlled Trial. *Obesity (Silver Spring, Md)*. 2020;doi:10.1002/oby.22980

117. John LK, Loewenstein G, Troxel AB, Norton L, Fassbender JE, Volpp KG. Financial incentives for extended weight loss: a randomized, controlled trial. *Journal of general internal medicine*. 2011;26(6):621-626. doi:10.1007/s11606-010-1628-y

118. Jolly K, Lewis A, Beach J, et al. Comparison of range of commercial or primary care led weight reduction programmes with minimal intervention control for weight loss in obesity: lighten Up randomised controlled trial. *BMJ: british medical journal (overseas & retired doctors edition)*. 2011;343(7832):1035-1035. doi:10.1136/bmj.d6500

119. Jospe MR, Roy M, Brown RC, et al. The Effect of Different Types of Monitoring Strategies on Weight Loss: a Randomized Controlled Trial. *Obesity (Silver Spring, Md)*. 2017;25(9):1490-1498. doi:10.1002/oby.21898

120. Juchacz K, Klos P, Dziedziejko V, Wojciak RW. The Effectiveness of Supportive Psychotherapy in Weight Loss in a Group of Young Overweight and Obese Women. *Nutrients*. 2021;13(2):06. doi:https://dx.doi.org/10.3390/nu13020532

121. Kaikkonen KM, Saltevo SS, Korpelainen JT, et al. Effective Weight Loss and Maintenance by Intensive Start with Diet and Exercise. *Medicine and science in sports and exercise*. 2019;51(5):920-929. doi:10.1249/MSS.0000000000001855

122. Kalarchian MA, Levine MD, Klem ML, Burke LE, Soulakova JN, Marcus MD. Impact of addressing reasons for weight loss on behavioral weight-control outcome. *American journal of preventive medicine*. 2011;40(1):18-24. doi:10.1016/j.amepre.2010.09.019

123. Keating SR, McCurry M. Text messaging as an intervention for weight loss in emerging adults. *Journal of the american association of nurse practitioners*. 2019;doi:10.1097/JXX.0000000000000176

124. Kempf K, Röhling M, Martin S, Schneider M. Telemedical coaching for weight loss in overweight employees: a three-armed randomised controlled trial. *BMJ open*. 2019;9(4):e022242. doi:10.1136/bmjopen-2018-022242

125. Kempf K, Rohling M, Stichert M, et al. Telemedical coaching improves long-term weight loss in overweight persons: a randomized controlled trial. *International journal of telemedicine and applications*. 2018;2018(no pagination)doi:10.1155/2018/7530602

126. Keogh JB, Pedersen E, Petersen KS, Clifton PM. Effects of intermittent compared to continuous energy restriction on short-term weight loss and long-term weight loss maintenance. *Clinical obesity*.

2014;4(3):150-156. doi:10.1111/cob.12052
127. Kim JY, Oh S, Steinhubl S, et al. Effectiveness of 6 months of tailored text message reminders for obese male participants in a worksite weight loss program: Randomized controlled trial. *JMIR mhealth and uhealth*. 2015;3(1)doi:10.2196/mhealth.3949
128. Kitzman H, Mamun A, Dodgen L, et al. Better Me Within Randomized Trial: faith-Based Diabetes Prevention Program for Weight Loss in African American Women. *American journal of health promotion*. 2020:890117120958545. doi:10.1177/0890117120958545
129. Knäuper B, Carrière K, Frayn M, et al. The Effects of If-Then Plans on Weight Loss: results of the McGill CHIP Healthy Weight Program Randomized Controlled Trial. *Obesity (Silver Spring, Md)*. 2018;26(8):1285-1295. doi:10.1002/oby.22226
130. Kraschnewski JL, Stuckey HL, Rovniak LS, et al. Efficacy of a weight-loss website based on positive deviance. A randomized trial. *American journal of preventive medicine*. 2011;41(6):610-614. doi:10.1016/j.amepre.2011.08.012
131. Krebs JD, Elley CR, Parry-Strong A, et al. The Diabetes Excess Weight Loss (DEWL) Trial: a randomised controlled trial of high-protein versus high-carbohydrate diets over 2 years in type 2 diabetes. *Diabetologia*. 2012;55(4):905-914. doi:10.1007/s00125-012-2461-0
132. Kumanyika SK, Fassbender JE, Sarwer DB, et al. One-year results of the Think Health! study of weight management in primary care practices. *Obesity (Silver Spring, Md)*. 2012;20(6):1249-1257. doi:10.1038/oby.2011.329
133. Laing BY, Mangione CM, Tseng CH, et al. Effectiveness of a smartphone application for weight loss compared with usual care in overweight primary care patients: a randomized, controlled trial. *Annals of internal medicine*. 2014;161(10 Suppl):S5-12. doi:10.7326/M13-3005
134. Latner JD, Ciao AC, Wendicke AU, Murakami JM, Durso LE. Community-based behavioral weight-loss treatment: long-term maintenance of weight loss, physiological, and psychological outcomes. *Behaviour research and therapy*. 2013;51(8):451-459. doi:10.1016/j.brat.2013.04.009
135. Leahey TM, Huedo-Medina TB, Grenga A, et al. Patient-provided e-support in reduced intensity obesity treatment: The INSPIRE randomized controlled trial. *Health psychology : official journal of the Division of Health Psychology, American Psychological Association*. 2020;39(12):1037-1047. doi:10.1037/hea0000996
136. Leahey TM, Subak LL, Fava J, et al. Benefits of adding small financial incentives or optional group meetings to a web-based statewide obesity initiative. *Obesity (Silver Spring, Md)*. 2015;23(1):70-76. doi:10.1002/oby.20937
137. Lerdrattanasakulchai Y, Palawisuth S. Do wristbands used as an adjunct to a weight- loss program affect the outcomes in the treatment of obesity? *Siriraj Medical Journal*. 2018;70(5):397-405. doi:10.14456/smj.2018.63
138. Lillis J, Dunsiger S, Thomas JG, Ross KM, Wing RR. Novel behavioral interventions to improve long-term weight loss: A randomized trial of acceptance and commitment therapy or self-regulation for weight loss maintenance. *Journal of Behavioral Medicine*. 2021;44(4):527-540. doi:10.1007/s10865-021-00215-z
139. Lin M, Mahmooth Z, Dedhia N, et al. Tailored, interactive text messages for enhancing weight loss among African American adults: the TRIMM randomized controlled trial. *American journal of medicine*. 2015;128(8):896-904. doi:10.1016/j.amjmed.2015.03.013
140. Linde JA, Simon GE, Ludman EJ, et al. A randomized controlled trial of behavioral weight loss treatment versus combined weight loss/depression treatment among women with comorbid obesity and depression. *Annals of behavioral medicine*. 2011;41(1):119-130. doi:10.1007/s12160-010-9232-2
141. Lison JF, Palomar G, Mensorio MS, et al. Impact of a Web-Based Exercise and Nutritional Education Intervention in Patients Who Are Obese with Hypertension: randomized Wait-List Controlled Trial. *Journal of medical Internet research*. 2020;22(4)doi:10.2196/14196
142. Little P, Stuart B, Hobbs FR, et al. An internet-based intervention with brief nurse support to manage obesity in primary care (POWeR+): a pragmatic, parallel-group, randomised controlled trial. *The lancet Diabetes & endocrinology*. 2016;4(10):821-828. doi:10.1016/S2213-8587(16)30099-7
143. Liu J, Godino JG, Norman GJ, et al. Planned care for obesity and cardiovascular risk reduction using a stepped-down approach: a randomized-controlled trial. *Preventive medicine*. 2018;114:223-231. doi:10.1016/j.ypmed.2018.07.015
144. Lockard B, Mardock M, Oliver JM, et al. Comparison of Two Diet and Exercise Approaches on Weight Loss and Health Outcomes in Obese Women. *International Journal of Environmental Research & Public Health [Electronic Resource]*. 2022;19(8):17. doi:https://dx.doi.org/10.3390/ijerph19084877
145. Loria-Kohen V, Gómez-Candela C, Fernández-Fernández C, Pérez-Torres A, García-Puig J, Bermejo LM. Evaluation of the usefulness of a low-calorie diet with or without bread in the treatment of overweight/obesity. *Clinical nutrition (Edinburgh, Scotland)*. 2012;31(4):455-461. doi:10.1016/j.clnu.2011.12.002
146. Luley C, Blaik A, Götz A, et al. Weight loss by telemonitoring of nutrition and physical activity in patients with metabolic syndrome for 1 year. *Journal of the American College of Nutrition*. 2014;33(5):363-374. doi:10.1080/07315724.2013.875437

147. Luley C, Blaik A, Reschke K, Klose S, Westphal S. Weight loss in obese patients with type 2 diabetes: effects of telemonitoring plus a diet combination - the Active Body Control (ABC) Program. *Diabetes research and clinical practice*. 2011;91(3):286-292. doi:10.1016/j.diabres.2010.11.020
148. Lynch EB, Liebman R, Ventrelle J, Avery EF, Richardson D. A self-management intervention for African Americans with comorbid diabetes and hypertension: a pilot randomized controlled trial. *Preventing chronic disease*. 2014;11:E90. doi:10.5888/pcd11.130349
149. Ma J, Strub P, Xiao L, Lavori PW, Camargo CA, Wilson SR. Behavioral weight loss and physical activity intervention in obese adults with asthma: a randomized trial. *Annals of the american thoracic society*. 2015;12(1):1-11.
150. Ma J, Yank V, Xiao L, et al. Translating the Diabetes Prevention Program lifestyle intervention for weight loss into primary care: a randomized trial. *JAMA internal medicine*. 2013;173(2):113-121. doi:10.1001/2013.jamainternmed.987
151. Madjd A, Taylor MA, Neek LS, et al. Effect of weekly physical activity frequency on weight loss in healthy overweight and obese women attending a weight loss program: a randomized controlled trial. *American journal of clinical nutrition*. 2016;104(5):1202-1208. doi:10.3945/ajcn.116.136408
152. Mangieri CW, Johnson RJ, Sweeney LB, Choi YU, Wood JC. Mobile health applications enhance weight loss efficacy following bariatric surgery. *Obesity research & clinical practice*. 2019;13(2):176-179. doi:10.1016/j.orcp.2019.01.004
153. Mansoori A, Khosravi M, Asadi M. Effects of evening meal timing on weight loss: a pilot open-labelled randomized clinical trial. *Journal of advances in medical and biomedical research*. 2020;28(126):41-46. doi:10.30699/jambs.28.126.41
154. Maroofi M, Nasrollahzadeh J. Effect of intermittent versus continuous calorie restriction on body weight and cardiometabolic risk markers in subjects with overweight or obesity and mild-to-moderate hypertriglyceridemia: a randomized trial. *Lipids in health and disease*. 2020;19(1)doi:10.1186/s12944-020-01399-0
155. Marrero DG, Palmer KN, Phillips EO, Miller-Kovach K, Foster GD, Saha CK. Comparison of Commercial and Self-Initiated Weight Loss Programs in People With Prediabetes: a Randomized Control Trial. *American journal of public health.* 2016;106(5):949-956. doi:10.2105/AJPH.2015.303035
156. Masheb RM, Grilo CM, Rolls BJ. A randomized controlled trial for obesity and binge eating disorder: low-energy-density dietary counseling and cognitive-behavioral therapy. *Behaviour research and therapy*. 2011;49(12):821-829. doi:10.1016/j.brat.2011.09.006
157. Mayer VL, Vangeepuram N, Fei K, et al. Outcomes of a Weight Loss Intervention to Prevent Diabetes Among Low-Income Residents of East Harlem, New York. *Health education & behavior*. 2019;46(6):1073-1082. doi:10.1177/1090198119868232
158. McDoniel SO, Hammond RS. A 24-week randomised controlled trial comparing usual care and metabolic-based diet plans in obese adults. *International journal of clinical practice*. 2010;64(11):1503-1511. doi:10.1111/j.1742-1241.2010.02464.x
159. McKee HC, Ntoumanis N. Developing self-regulation for dietary temptations: intervention effects on physical, self-regulatory and psychological outcomes. *Journal of behavioral medicine*. 2014;37(6):1075-1081. doi:10.1007/s10865-014-9557-6
160. McLeod M, Stanley J, Signal V, et al. Impact of a comprehensive digital health programme on HbA1c and weight after 12 months for people with diabetes and prediabetes: a randomised controlled trial. *Diabetologia*. 2020;63(12):2559-2570. doi:10.1007/s00125-020-05261-x
161. McRobbie H, Hajek P, Peerbux S, et al. Tackling obesity in areas of high social deprivation: clinical effectiveness and cost-effectiveness of a task-based weight management group programme - A randomised controlled trial and economic evaluation. *Health technology assessment*. 2016;20(79) (no pagination)doi:10.3310/hta20790
162. McRobbie H, Hajek P, Peerbux S, et al. Randomised controlled trial and economic evaluation of a task-based weight management group programme. *BMC public health*. 2019;19(1):365. doi:10.1186/s12889-019-6679-3
163. Mehring M, Haag M, Linde K, et al. Effects of a general practice guided web-based weight reduction program--results of a cluster-randomized controlled trial. *BMC family practice*. 2013;14:76. doi:10.1186/1471-2296-14-76
164. Melanson KJ, Summers A, Nguyen V, et al. Body composition, dietary composition, and components of metabolic syndrome in overweight and obese adults after a 12-week trial on dietary treatments focused on portion control, energy density, or glycemic index. *Nutrition journal*. 2012;11(1)doi:10.1186/1475-2891-11-57
165. Melchart D, Low P, Wuhr E, Kehl V, Weidenhammer W. Effects of a tailored lifestyle self-management intervention (TALENT) study on weight reduction: a randomized controlled trial. *Diabetes, metabolic syndrome and obesity: targets and therapy*. 2017;10:235-245. doi:10.2147/DMSO.S135572
166. Miller CK, Kristeller JL, Headings A, Nagaraja H. Comparison of a mindful eating intervention to a diabetes self-management intervention among adults with type 2 diabetes: a randomized controlled trial. *Health*

education & behavior. 2014;41(2):145-154. doi:10.1177/1090198113493092

167. Milsom VA, Malcolm RJ, Johnson GC, et al. Changes in cardiovascular risk factors with participation in a 12-week weight loss trial using a commercial format. *Eating behaviors*. 2014;15(1):68-71. doi:10.1016/j.eatbeh.2013.10.004
168. Mishra S, Xu J, Agarwal U, Gonzales J, Levin S, Barnard ND. A multicenter randomized controlled trial of a plant-based nutrition program to reduce body weight and cardiovascular risk in the corporate setting: The GEICO study. *European journal of clinical nutrition*. 2013;67(7):718-724. doi:10.1038/ejcn.2013.92
169. Mohamad H, Ntessalen M, Craig LCA, et al. A self-help diet and physical activity intervention with dietetic support for weight management in men treated for prostate cancer: pilot study of the Prostate Cancer Weight Management (PRO-MAN) randomised controlled trial. *British journal of nutrition*. 2019;122(5):592-600. doi:10.1017/S0007114519001090
170. Moncrieft AE, Llabre MM, McCalla JR, et al. Effects of a Multicomponent Life-Style Intervention on Weight, Glycemic Control, Depressive Symptoms, and Renal Function in Low-Income, Minority Patients With Type 2 Diabetes: results of the Community Approach to Lifestyle Modification for Diabetes Randomized Controlled Trial. *Psychosomatic medicine*. 2016;78(7):851-860. doi:10.1097/PSY.0000000000000348
171. Moravcová K, Karbanová M, Bretschneider MP, Sovová M, Ožana J, Sovová E. Comparing Digital Therapeutic Intervention with an Intensive Obesity Management Program: Randomized Controlled Trial. *Nutrients*. 2022;14(10)doi:10.3390/nu14102005
172. Morgan PJ, Collins CE, Plotnikoff RC, et al. Efficacy of a workplace-based weight loss program for overweight male shift workers: the Workplace POWER (Preventing Obesity Without Eating like a Rabbit) randomized controlled trial. *Preventive medicine*. 2011;52(5):317-325. doi:10.1016/j.ypmed.2011.01.031
173. Morgan PJ, Collins CE, Plotnikoff RC, et al. The impact of a workplace-based weight loss program on work-related outcomes in overweight male shift workers. *Journal of occupational and environmental medicine*. 2012;54(2):122-127. doi:10.1097/JOM.0b013e31824329ab
174. Moss EL, Tobin LN, Campbell TS, von Ranson KM. Behavioral weight-loss treatment plus motivational interviewing versus attention control: lessons learned from a randomized controlled trial. *Trials*. 2017;18(1):351. doi:10.1186/s13063-017-2094-1
175. Nakata Y, Okada M, Hashimoto K, Harada Y, Sone H, Tanaka K. Comparison of education-only versus group-based intervention in promoting weight loss: a randomised controlled trial. *Obesity facts*. 2011;4(3):222-228. doi:10.1159/000329619
176. Nakata Y, Sasai H, Tsujimoto T, Hashimoto K, Kobayashi H. A Single Motivational Lecture Can Promote Modest Weight Loss: a Randomized Controlled Trial. *Obesity facts*. 2020;13(2):267-278. doi:10.1159/000506813
177. Nanchahal K, Power T, Holdsworth E, Hession M, Sorhaindo A, Griffiths U. A pragmatic randomised controlled trial in primary care of the Camden Weight Loss (CAMWEL) programme. *BMJ open*. 2012;2(3)doi:10.1136/bmjopen-2011-000793
178. Napolitano MA, Whiteley JA, Mavredes M, et al. Effect of tailoring on weight loss among young adults receiving digital interventions: an 18 month randomized controlled trial. *Translational Behavioral Medicine*. 2021;11(4):970-980. doi:https://dx.doi.org/10.1093/tbm/ibab017
179. Neumeier WH, Guerra N, Hsieh K, Thirumalai M, Ervin D, Rimmer JH. POWERSforID: Personalized online weight and exercise response system for individuals with intellectual disability: A randomized controlled trial. *Disability & Health Journal*. 2021;14(4):101111. doi:https://dx.doi.org/10.1016/j.dhjo.2021.101111
180. Newton RL, Carter LA, Johnson W, et al. A Church-Based Weight Loss Intervention in African American Adults using Text Messages (LEAN Study): cluster Randomized Controlled Trial. *Journal of medical Internet research*. 2018;20(8):e256. doi:10.2196/jmir.9816
181. Nezami BT, Hurley L, Power J, Valle CG, Tate DF. A pilot randomized trial of simplified versus standard calorie dietary self-monitoring in a mobile weight loss intervention. *Obesity*. 2022;30(3):628-638. doi:10.1002/oby.23377
182. Nilsen V, Bakke PS, Gallefoss F. Effects of lifestyle intervention in persons at risk for type 2 diabetes mellitus - results from a randomised, controlled trial. *BMC public health*. 2011;11:893. doi:10.1186/1471-2458-11-893
183. Noreik M, Madigan CD, Astbury NM, et al. Testing the short-term effectiveness of primary care referral to online weight loss programmes: A randomised controlled trial. *Clinical Obesity*. 2021;11(6):e12482. doi:https://dx.doi.org/10.1111/cob.12482
184. Nybacka Å, Carlström K, Ståhle A, Nyrén S, Hellström PM, Hirschberg AL. Randomized comparison of the influence of dietary management and/or physical exercise on ovarian function and metabolic parameters in overweight women with polycystic ovary syndrome. *Fertility and sterility*. 2011;96(6):1508-1513. doi:10.1016/j.fertnstert.2011.09.006
185. Oh B, Cho B, Han MK, et al. The effectiveness of mobile phone-based care for weight control in metabolic syndrome patients: Randomized controlled trial. *JMIR mhealth and uhealth*. 2015;3(3)doi:10.2196/mhealth.4222
186. Olson R, Wipfli B, Thompson SV, et al. Weight Control Intervention for Truck Drivers: the SHIFT

Randomized Controlled Trial, United States. *American journal of public health*. 2016;106(9):1698-1706. doi:10.2105/AJPH.2016.303262
187. Østbye T, Stroo M, Brouwer RJ, et al. Steps to Health employee weight management randomized control trial: short-term follow-up results. *Journal of occupational and environmental medicine*. 2015;57(2):188-195. doi:10.1097/JOM.0000000000000335
188. Ozaki I, Watai I, Nishijima M, Saito N. Randomized controlled trial of Web-based weight-loss intervention with human support for male workers under 40. *Journal of occupational health*. 2019;61(1):110-120. doi:10.1002/1348-9585.12037
189. Padwal RS, Klarenbach S, Sharma AM, et al. The evaluating self-management and educational support in severely obese patients awaiting multidisciplinary bariatric care (EVOLUTION) trial: principal results. *BMC medicine*. 2017;15(1):46. doi:10.1186/s12916-017-0808-6
190. Pagoto S, Schneider KL, Whited MC, et al. Randomized controlled trial of behavioral treatment for comorbid obesity and depression in women: the Be Active Trial. *International journal of obesity (2005)*. 2013;37(11):1427-1434. doi:10.1038/ijo.2013.25
191. Palavras MA, Hay P, Mannan H, et al. Integrated weight loss and cognitive behavioural therapy (CBT) for the treatment of recurrent binge eating and high body mass index: a randomized controlled trial. *Eating and weight disorders*. 2020;doi:10.1007/s40519-020-00846-2
192. Patel ML, Hopkins CM, Brooks TL, Bennett GG. Comparing Self-Monitoring Strategies for Weight Loss in a Smartphone App: randomized Controlled Trial. *JMIR mhealth and uhealth*. 2019;7(2):e12209. doi:10.2196/12209
193. Patrick K, Calfas KJ, Norman GJ, et al. Outcomes of a 12-month web-based intervention for overweight and obese men. *Annals of behavioral medicine*. 2011;42(3):391-401. doi:10.1007/s12160-011-9296-7
194. Perri MG, Limacher MC, von Castel-Roberts K, et al. Comparative effectiveness of three doses of weight-loss counseling: two-year findings from the rural LITE trial. *Obesity (Silver Spring, Md)*. 2014;22(11):2293-2300. doi:10.1002/oby.20832
195. Poelman MP, de Vet E, Velema E, de Boer MR, Seidell JC, Steenhuis IHM. PortionControl@HOME: Results of a Randomized Controlled Trial Evaluating the Effect of a Multi-Component Portion Size Intervention on Portion Control Behavior and Body Mass Index. *Annals of behavioral medicine*. 2015;49(1):18-28. doi:10.1007/s12160-014-9637-4
196. Psota TL, Tindall AM, Lohse B, Miller PE, Petersen KS, Kris-Etherton PM. The Weight Optimization Revamping Lifestyle using the Dietary Guidelines (WORLD) Study: sustained Weight Loss Over 12 Months. *Obesity (Silver Spring, Md)*. 2020;28(7):1235-1244. doi:10.1002/oby.22824
197. Ratliff JC, Palmese LB, Tonizzo KM, Chwastiak L, Tek C. Contingency management for the treatment of antipsychotic-induced weight gain: a randomized controlled pilot study. *Obesity facts*. 2015;5(6):919-927. doi:10.1159/000345975
198. Reeves M, Winkler E, McCarthy N, et al. The Living Well after Breast Cancer™ Pilot Trial: a weight loss intervention for women following treatment for breast cancer. *Asia-Pacific journal of clinical oncology*. 2017;13(3):125-136. doi:10.1111/ajco.12629
199. Rosas LG, Lv N, Xiao L, et al. Effect of a Culturally Adapted Behavioral Intervention for Latino Adults on Weight Loss Over 2 Years: A Randomized Clinical Trial. *JAMA network open*. 2020;3(12):e2027744. doi:10.1001/jamanetworkopen.2020.27744
200. Rosas LG, Thiyagarajan S, Goldstein BA, et al. The effectiveness of two community-based weight loss strategies among obese, low-income US Latinos. *Journal of the academy of nutrition and dietetics*. 2015;115(4):537-50.e2. doi:10.1016/j.jand.2014.10.020
201. Rosas LG, Vasquez JJ, Hedlin HK, et al. Comparing enhanced versus standard Diabetes Prevention Program among indigenous adults in an urban setting: a randomized controlled trial. *BMC public health*. 2020;20(1):139. doi:10.1186/s12889-020-8250-7
202. Ross KM, Wing RR. Impact of newer self-monitoring technology and brief phone-based intervention on weight loss: a randomized pilot study. *Obesity (Silver Spring, Md)*. 2016;24(8):1653-1659. doi:10.1002/oby.21536
203. Rounds T, Crane M, Harvey J. The Impact of Incentives on Weight Control in Men: a Randomized Controlled Trial. *American journal of men's health*. 2020;14(1):1557988319895147. doi:10.1177/1557988319895147
204. Saffari M, Pakpour AH, Mohammadi-Zeidi I, Samadi M, Chen H. Long-Term Effect of Motivational Interviewing on Dietary Intake and Weight Loss in Iranian Obese/Overweight Women. *Health promotion perspectives*. 2014;4(2):206-213. doi:10.5681/hpp.2014.027
205. Samuel-Hodge CD, Garcia BA, Johnston LF, et al. Translation of a behavioral weight loss intervention for mid-life, low-income women in local health departments. *Obesity (Silver Spring, Md)*. 2013;21(9):1764-1773. doi:10.1002/oby.20317
206. Samuel-Hodge CD, Holder-Cooper JC, Gizlice Z, et al. Family PArtners in Lifestyle Support (PALS): family-based weight loss for African American adults with type 2 diabetes. *Obesity (Silver Spring, Md)*. 2017;25(1):45-55. doi:10.1002/oby.21700

207. Sant'Anna EM, Paiva SPC, Santos RP, et al. Mindfulness-based program to support lifestyle modification and weight loss in infertile women: randomized controlled trial. *Journal of Psychosomatic Obstetrics and Gynecology*. 2022;43(2):136-144. doi:10.1080/0167482X.2020.1823962
208. Santa-Maria CA, Coughlin JW, Sharma D, et al. The Effects of a Remote-Based Weight Loss Program on Adipocytokines, Metabolic Markers, and Telomere Length in Breast Cancer Survivors: the POWER-Remote Trial. *Clinical cancer research*. 2020;doi:10.1158/1078-0432.CCR-19-2935
209. Satoshi K, Takumi K, Takasawa N, Yukiko M, Atsushi I, Ryuki K. Interventions for body weight reduction in obese patients during short consultations: an open-label randomized controlled trial in the Japanese primary care setting. *Asia Pacific family medicine*. 2015;14:1-12. doi:10.1186/s12930-015-0022-7
210. Seif-Barghi T, Akbari-Fakhrabadi M, Teimori MP, Tashk A, Alizadeh Z, Memari AH. Cognitive Behavior Therapy's Effect in a Weight Loss Program Among Obese Iranian Women. *Nutrition today*. 2018;53(4):174-178. doi:10.1097/NT.0000000000000281
211. Sellman D, Schroder R, Deering D, Elmslie J, Foulds J, Frampton C. Psychosocial enhancement of the Green Prescription for obesity recovery: a randomised controlled trial. *New Zealand medical journal*. 2017;130(1450):44-54.
212. Shapiro JR, Koro T, Doran N, et al. Text4Diet: a randomized controlled study using text messaging for weight loss behaviors. *Preventive medicine*. 2012;55(5):412-417. doi:10.1016/j.ypmed.2012.08.011
213. Shuger SL, Barry VW, Sui X, et al. Electronic feedback in a diet- and physical activity-based lifestyle intervention for weight loss: a randomized controlled trial. *International journal of behavioral nutrition and physical activity*. 2011;8:41. doi:10.1186/1479-5868-8-41
214. Silina V, Tessma MK, Senkane S, Krievina G, Bahs G. Text messaging (SMS) as a tool to facilitate weight loss and prevent metabolic deterioration in clinically healthy overweight and obese subjects: a randomised controlled trial. *Scandinavian journal of primary health care*. 2017;35(3):262-270. doi:10.1080/02813432.2017.1358435
215. Smith JR, Greaves CJ, Thompson JL, et al. The community-based prevention of diabetes (ComPoD) study: a randomised, waiting list controlled trial of a voluntary sector-led diabetes prevention programme. *International journal of behavioral nutrition and physical activity*. 2019;16(1):N.PAG. doi:10.1186/s12966-019-0877-3
216. Spadaro KC, Davis KK, Sereika SM, Gibbs BB, Jakicic JM, Cohen SM. Effect of mindfulness meditation on short-term weight loss and eating behaviors in overweight and obese adults: a randomized controlled trial. *Journal of complementary & integrative medicine*. 2018;15(2):N.PAG. doi:10.1515/jcim-2016-0048
217. Spring B, Duncan JM, Janke EA, et al. Integrating technology into standard weight loss treatment: a randomized controlled trial. *JAMA internal medicine*. 2013;173(2):105-111. doi:10.1001/jamainternmed.2013.1221
218. Stephens JD, Yager AM, Allen J. Smartphone Technology and Text Messaging for Weight Loss in Young Adults: a Randomized Controlled Trial. *Journal of cardiovascular nursing*. 2017;32(1):39-46. doi:10.1097/JCN.0000000000000307
219. Stolley M, Sheean P, Gerber B, et al. Efficacy of a weight loss intervention for african American breast cancer survivors. *Journal of clinical oncology*. 2017;35(24):2820-2828. doi:10.1200/JCO.2016.71.9856
220. Sugawara N, Sagae T, Yasui-Furukori N, et al. Effects of nutritional education on weight change and metabolic abnormalities among patients with schizophrenia in Japan: a randomized controlled trial. *Journal of psychiatric research*. 2018;97:77-83. doi:10.1016/j.jpsychires.2017.12.002
221. Suminski RR, Leonard T, Obrusnikova I, Kelly K. The Impact of Health Coaching on Weight and Physical Activity in Obese Adults: A Randomized Control Trial. *American Journal of Lifestyle Medicine*. 2022;doi:10.1177/15598276221114047
222. Sundfor TM, Svendsen M, Tonstad S. Effect of intermittent versus continuous energy restriction on weight loss, maintenance and cardiometabolic risk: a randomized 1-year trial. *Nutrition, metabolism and cardiovascular diseases*. 2018;(no pagination)doi:10.1016/j.numecd.2018.03.009
223. Svetkey LP, Batch BC, Lin PH, et al. Cell phone intervention for you (CITY): a randomized, controlled trial of behavioral weight loss intervention for young adults using mobile technology. *Obesity (Silver Spring, Md)*. 2015;23(11):2133-2141. doi:10.1002/oby.21226
224. Swisher AK, Abraham J, Bonner D, et al. Exercise and dietary advice intervention for survivors of triple-negative breast cancer: effects on body fat, physical function, quality of life, and adipokine profile. *Supportive care in cancer*. 2015;23(10):2995-3003. doi:10.1007/s00520-015-2667-z
225. Tanaka K, Sasai H, Wakaba K, et al. Professional dietary coaching within a group chat using a smartphone application for weight loss: a randomized controlled trial. *Journal of multidisciplinary healthcare*. 2018;11:339-347. doi:10.2147/JMDH.S165422
226. Tate DF, Lutes LD, Bryant M, et al. Efficacy of a Commercial Weight Management Program Compared With a Do-It-Yourself Approach: A Randomized Clinical Trial. *JAMA Network Open*. 2022;5(8):e2226561. doi:https://dx.doi.org/10.1001/jamanetworkopen.2022.26561
227. Teeriniemi AM, Salonurmi T, Jokelainen T, et al. A randomized clinical trial of the effectiveness of a Web-based health behaviour change support system and group lifestyle counselling on body weight loss in

overweight and obese subjects: 2-year outcomes. *Journal of internal medicine*. 2018;284(5):534-545. doi:10.1111/joim.12802
228. Tejera C, Porca C, Rodriguez-Carnero G, et al. Reducing Metabolic Syndrome through a Group Educational Intervention Program in Adults with Obesity: IGOBE Program. *Nutrients*. 2022;14(5):03. doi:https://dx.doi.org/10.3390/nu14051066
229. Thomas JG, Bond DS, Raynor HA, Papandonatos GD, Wing RR. Comparison of Smartphone-Based Behavioral Obesity Treatment With Gold Standard Group Treatment and Control: a Randomized Trial. *Obesity (Silver Spring, Md)*. 2019;doi:10.1002/oby.22410
230. Thomas JG, Leahey TM, Wing RR. An automated internet behavioral weight-loss program by physician referral: a randomized controlled trial. *Diabetes care*. 2015;38(1):9-15. doi:10.2337/dc14-1474
231. Thomas JG, Raynor HA, Bond DS, et al. Weight loss in Weight Watchers Online with and without an activity tracking device compared to control: a randomized trial. *Obesity (Silver Spring, Md)*. 2017;25(6):1014-1021. doi:10.1002/oby.21846
232. Thomas JG, Raynor HA, Bond DS, et al. Weight loss and frequency of body-weight self-monitoring in an online commercial weight management program with and without a cellular-connected 'smart' scale: a randomized pilot study. *Obesity science and practice*. 2017;3(4):365-372. doi:10.1002/osp4.132
233. Tsai AG, Wadden TA, Rogers MA, Day SC, Moore RH, Islam BJ. A primary care intervention for weight loss: results of a randomized controlled pilot study. *Obesity (Silver Spring, Md)*. 2010;18(8):1614-1618. doi:10.1038/oby.2009.457
234. Turner-McGrievy G, Tate D. Tweets, Apps, and Pods: results of the 6-month Mobile Pounds Off Digitally (Mobile POD) randomized weight-loss intervention among adults. *Journal of medical Internet research*. 2011;13(4):e120. doi:10.2196/jmir.1841
235. Turner-McGrievy GM, Davidson CR, Wingard EE, Billings DL. Low glycemic index vegan or low-calorie weight loss diets for women with polycystic ovary syndrome: a randomized controlled feasibility study. *Nutrition research (New York, NY)*. 2014;34(6):552-558. doi:10.1016/j.nutres.2014.04.011
236. Turner-McGrievy GM, Davidson CR, Wingard EE, Wilcox S, Frongillo EA. Comparative effectiveness of plant-based diets for weight loss: a randomized controlled trial of five different diets. *Nutrition (Burbank, Los Angeles County, Calif)*. 2015;31(2):350-358. doi:10.1016/j.nut.2014.09.002
237. Turner-McGrievy GM, Wilcox S, Boutté A, et al. The Dietary Intervention to Enhance Tracking with Mobile Devices (DIET Mobile) Study: a 6-Month Randomized Weight Loss Trial. *Obesity (Silver Spring, Md)*. 2017;25(8):1336-1342. doi:10.1002/oby.21889
238. Unick JL, Dunsiger SI, Bock BC, Sherman SA, Braun TD, Wing RR. A preliminary investigation of yoga as an intervention approach for improving long-term weight loss: A randomized trial. *PLoS ONE [Electronic Resource]*. 2022;17(2):e0263405. doi:https://dx.doi.org/10.1371/journal.pone.0263405
239. van Wier MF, Dekkers JC, Hendriksen IJ, et al. Effectiveness of phone and e-mail lifestyle counseling for long term weight control among overweight employees. *Journal of occupational and environmental medicine*. 2011;53(6):680-686. doi:10.1097/JOM.0b013e31821f2bbb
240. Vaz CL, Carnes N, Pousti B, Zhao H, Williams KJ. A randomized controlled trial of an innovative, user-friendly, interactive smartphone app-based lifestyle intervention for weight loss. *Obesity Science and Practice*. 2021;7(5):555-568. doi:10.1002/osp4.503
241. Venditti EM, Marcus MD, Miller RG, Arena VC, Greenspan SL, Rockette-Wagner B. Group Lifestyle Phone Maintenance for Weight, Health, and Physical Function in Adults Aged 65-80 Years: A Randomized Clinical Trial. *Journals of Gerontology Series A-Biological Sciences & Medical Sciences*. 2021;76(2):352-360. doi:https://dx.doi.org/10.1093/gerona/glaa229
242. Ventura Marra M, Lilly CL, Nelson KR, Woofter DR, Malone J. A Pilot Randomized Controlled Trial of a Telenutrition Weight Loss Intervention in Middle-Aged and Older Men with Multiple Risk Factors for Cardiovascular Disease. *Nutrients*. 2019;11(2)doi:10.3390/nu11020229
243. von Gruenigen V, Frasure H, Kavanagh MB, et al. Survivors of uterine cancer empowered by exercise and healthy diet (SUCCEED): a randomized controlled trial. *Gynecologic oncology*. 2012;125(3):699-704. doi:10.1016/j.ygyno.2012.03.042
244. Watson S, Woodside JV, Ware LJ, et al. Effect of a Web-Based Behavior Change Program on Weight Loss and Cardiovascular Risk Factors in Overweight and Obese Adults at High Risk of Developing Cardiovascular Disease: randomized Controlled Trial. *Journal of medical Internet research*. 2015;17(7):e177. doi:10.2196/jmir.3828
245. Webber KH, Gabriele JM, Tate DF, Dignan MB. The effect of a motivational intervention on weight loss is moderated by level of baseline controlled motivation. *International journal of behavioral nutrition and physical activity*. 2010;7:4. doi:10.1186/1479-5868-7-4
246. Wennehorst K, Mildenstein K, Saliger B, et al. A Comprehensive Lifestyle Intervention to Prevent Type 2 Diabetes and Cardiovascular Diseases: the German CHIP Trial. *Prevention science*. 2016;17(3):386-397. doi:10.1007/s11121-015-0623-2
247. West DS, Bursac Z, Cornell CE, et al. Lay health educators translate a weight-loss intervention in senior centers: a randomized controlled trial. *American journal of preventive medicine*. 2011;41(4):385-391.

doi:10.1016/j.amepre.2011.06.041
248. West DS, Harvey JR, Krukowski RA, Prewitt TE, Priest J, Ashikaga T. Do individual, online motivational interviewing chat sessions enhance weight loss in a group-based, online weight control program? *Obesity (Silver Spring, Md)*. 2016;24(11):2334-2340. doi:10.1002/oby.21645
249. West DS, Krukowski RA, Finkelstein EA, et al. Adding Financial Incentives to Online Group-Based Behavioral Weight Control: an RCT. *American journal of preventive medicine*. 2020;doi:10.1016/j.amepre.2020.03.015
250. Whitelock V, Kersbergen I, Higgs S, Aveyard P, Halford JCG, Robinson E. A smartphone based attentive eating intervention for energy intake and weight loss: results from a randomised controlled trial. *BMC public health*. 2019;19(1):611. doi:10.1186/s12889-019-6923-x
251. Wright N, Wilson L, Smith M, Duncan B, McHugh P. The BROAD study: a randomised controlled trial using a whole food plant-based diet in the community for obesity, ischaemic heart disease or diabetes. *Nutrition & diabetes*. 2017;7(3):e256. doi:10.1038/nutd.2017.3
252. Yancy WS, Shaw PA, Reale C, et al. Effect of Escalating Financial Incentive Rewards on Maintenance of Weight Loss: a Randomized Clinical Trial. *JAMA network open*. 2019;2(11):e1914393. doi:10.1001/jamanetworkopen.2019.14393
253. Yeary KHK, Cornell CE, Moore PC, Gauss CH, Prewitt TE, Turner J. The WORD: outcomes of a Behavioral Weight Loss Maintenance Effectiveness Trial in Rural Black Adults of Faith. *Obesity (Silver Spring, Md)*. 2020;28(3):510-520. doi:10.1002/oby.22717
254. Young MD, Drew RJ, Kay-Lambkin F, et al. Impact of a self-guided, eHealth program targeting weight loss and depression in men: A randomized trial. *Journal of Consulting & Clinical Psychology*. 2021;89(8):682-694. doi:https://dx.doi.org/10.1037/ccp0000671
255. Zamorano AS, Wilson EM, Liu J, et al. Text-message-based behavioral weight loss for endometrial cancer survivors with obesity: A randomized controlled trial. *Gynecologic Oncology*. 2021;162(3):770-777. doi:https://dx.doi.org/10.1016/j.ygyno.2021.06.007
256. Zinn C, McPhee J, Harris N, Williden M, Prendergast K, Schofield G. A 12-week low-carbohydrate, high-fat diet improves metabolic health outcomes over a control diet in a randomised controlled trial with overweight defence force personnel. *Physiologie appliquee, nutrition ET metabolisme [applied physiology, nutrition, and metabolism]*. 2017;42(11):1158-1164. doi:10.1139/apnm-2017-0260
257. Zwickert K, Rieger E, Swinbourne J, et al. High or low intensity text-messaging combined with group treatment equally promote weight loss maintenance in obese adults. *Obesity research & clinical practice*. 2016;10(6):680-691. doi:10.1016/j.orcp.2016.01.001

Supplementary B: PRISMA Checklist

Section and Topic	Item #	Checklist item	Location where item is reported
TITLE			
Title	1	Identify the report as a systematic review.	Title Page, Page 1, Line 1
ABSTRACT			
Abstract	2	See the PRISMA 2020 for Abstracts checklist.	Page 3, Line 30-54
INTRODUCTION			
Rationale	3	Describe the rationale for the review in the context of existing knowledge.	Page 4-5, Line
Objectives	4	Provide an explicit statement of the objective(s) or question(s) the review addresses.	Page 5, Line 84-89
METHODS			
Eligibility criteria	5	Specify the inclusion and exclusion criteria for the review and how studies were grouped for the syntheses.	Page 6, Line 100-118
Information sources	6	Specify all databases, registers, websites, organisations, reference lists and other sources searched or consulted to identify studies. Specify the date when each source was last searched or consulted.	Page 5, Line 94-99
Search strategy	7	Present the full search strategies for all databases, registers and websites, including any filters and limits used.	Supplementary 2
Selection process	8	Specify the methods used to decide whether a study met the inclusion criteria of the review, including how many reviewers screened each record and each report retrieved, whether they worked independently, and if applicable, details of automation tools used in the process.	Page 19, Line 119-127
Data collection process	9	Specify the methods used to collect data from reports, including how many reviewers collected data from each report, whether they worked independently, any processes for obtaining or confirming data from study investigators, and if applicable, details of automation tools used in the process.	Page 20, Line 128-136
Data items	10a	List and define all outcomes for which data were sought. Specify whether all results that were compatible with each outcome domain in each study were sought (e.g. for all measures, time points, analyses), and if not, the methods used to decide which results to collect.	Page 8, Table 1
	10b	List and define all other variables for which data were sought (e.g. participant and intervention characteristics, funding sources). Describe any assumptions made about any missing or unclear information.	Page 8, Table 1
Study risk of bias assessment	11	Specify the methods used to assess risk of bias in the included studies, including details of the tool(s) used, how many reviewers assessed each study and whether they worked independently, and if applicable, details of automation tools used in the process.	Page 7, Line 148-149
Effect measures	12	Specify for each outcome the effect measure(s) (e.g. risk ratio, mean difference) used in the synthesis or presentation of results.	Page 7, Line 137-148
Synthesis methods	13a	Describe the processes used to decide which studies were eligible for each synthesis (e.g. tabulating the study intervention characteristics and comparing against the planned groups for each synthesis (item #5)).	Page 7, Line 137-148
	13b	Describe any methods required to prepare the data for presentation or synthesis, such as handling of missing summary statistics, or data conversions.	Page 7, Line 137-148

Supplementary B: PRISMA Checklist

Section and Topic	Item #	Checklist item	Location where item is reported
	13c	Describe any methods used to tabulate or visually display results of individual studies and syntheses.	Page 7, Line 137-148
	13d	Describe any methods used to synthesize results and provide a rationale for the choice(s). If meta-analysis was performed, describe the model(s), method(s) to identify the presence and extent of statistical heterogeneity, and software package(s) used.	Page 7, Line 137-148
	13e	Describe any methods used to explore possible causes of heterogeneity among study results (e.g. subgroup analysis, meta-regression).	N/A
	13f	Describe any sensitivity analyses conducted to assess robustness of the synthesized results.	N/A
Reporting bias assessment	14	Describe any methods used to assess risk of bias due to missing results in a synthesis (arising from reporting biases).	N/A
Certainty assessment	15	Describe any methods used to assess certainty (or confidence) in the body of evidence for an outcome.	N/A
RESULTS			
Study selection	16a	Describe the results of the search and selection process, from the number of records identified in the search to the number of studies included in the review, ideally using a flow diagram.	Figure 1
	16b	Cite studies that might appear to meet the inclusion criteria, but which were excluded, and explain why they were excluded.	Figure 1
Study characteristics	17	Cite each included study and present its characteristics.	Table 2<br>Page 9, Line 157-158, 159-169
Risk of bias in studies	18	Present assessments of risk of bias for each included study.	N/A
Results of individual studies	19	For all outcomes, present, for each study: (a) summary statistics for each group (where appropriate) and (b) an effect estimate and its precision (e.g. confidence/credible interval), ideally using structured tables or plots.	N/A
Results of syntheses	20a	For each synthesis, briefly summarise the characteristics and risk of bias among contributing studies.	Page 11-12, Line 172-216
	20b	Present results of all statistical syntheses conducted. If meta-analysis was done, present for each the summary estimate and its precision (e.g. confidence/credible interval) and measures of statistical heterogeneity. If comparing groups, describe the direction of the effect.	Page 11-12, Line 172-216
	20c	Present results of all investigations of possible causes of heterogeneity among study results.	Page 11-12, Line 185-210
	20d	Present results of all sensitivity analyses conducted to assess the robustness of the synthesized results.	N/A
Reporting biases	21	Present assessments of risk of bias due to missing results (arising from reporting biases) for each synthesis assessed.	N/A
Certainty of evidence	22	Present assessments of certainty (or confidence) in the body of evidence for each outcome assessed.	N/A
DISCUSSION			

Supplementary B: PRISMA Checklist

Section and Topic	Item #	Checklist item	Location where item is reported
Discussion	23a	Provide a general interpretation of the results in the context of other evidence.	Page 14-19, Line 236-361
	23b	Discuss any limitations of the evidence included in the review.	Page 19, Line 369-377
	23c	Discuss any limitations of the review processes used.	Page 19, Line 369-377
	23d	Discuss implications of the results for practice, policy, and future research.	Page 19-20, Line 378-400
OTHER INFORMATION			
Registration and protocol	24a	Provide registration information for the review, including register name and registration number, or state that the review was not registered.	Methods, Page 5, Line 92-93
	24b	Indicate where the review protocol can be accessed, or state that a protocol was not prepared.	N/A
	24c	Describe and explain any amendments to information provided at registration or in the protocol.	N/A
Support	25	Describe sources of financial or non-financial support for the review, and the role of the funders or sponsors in the review.	Title page, Page 1, Line 22-23
Competing interests	26	Declare any competing interests of review authors.	Title page, Page 1, Line 27
Availability of data, code and other materials	27	Report which of the following are publicly available and where they can be found: template data collection forms; data extracted from included studies; data used for all analyses; analytic code; any other materials used in the review.	Title page, Page 2, Line 28-29

From: Page MJ, McKenzie JE, Bossuyt PM, Boutron I, Hoffmann TC, Mulrow CD, et al. The PRISMA 2020 statement: an updated guideline for reporting systematic reviews. BMJ 2021;372:n71. doi: 10.1136/bmj.n71. This work is licensed under CC BY 4.0. To view a copy of this license, visit https://creativecommons.org/licenses/by/4.0/

Supplementary C: Search Strategy

The following tables indicate the database and search terms.

**Database: SCOPUS**		
#	**Search Terms**	**Fields**
1	"weight loss"	Title, Abstract, Keywords
2	random*	Title, Abstract, Keywords
3	RCT	Title, Abstract, Keywords
4	1 AND (2 OR 3)	
5	Limit to: Article; Humans; Adults; English language	Filters

**Database: CENTRAL**		
#	**Search Terms**	**Fields**
1	"weight loss"	Title, Abstract, Keywords
2	random*	Title, Abstract, Keywords
3	RCT	Title, Abstract, Keywords
4	1 AND (2 OR 3)	
5	Limit to: Trials (CENTRAL default), Adults; English language	Filters

**Database: Medline**		
#	**Search Terms**	**Fields**
1	"weight loss"	Title/Abstract
2	random*	Title/Abstract
3	RCT OR "randomized controlled trial"	Title/Abstract / Publication Type
4	1 AND (2 OR 3)	
5	Limit to: Humans; English language; Adults (18+)	Filters
